# Engineering the Host Defense Peptide from Scorpion Venom for Safer and More Potent Antibreast Cancer Activity

**DOI:** 10.1021/acsptsci.5c00598

**Published:** 2026-05-05

**Authors:** Cyntia Silva Oliveira, Laertty Garcia Sousa Cabral, Rosely Cabette Barbosa Alves, Gislaine Patricia Andrade, Anderson Orzari Ribeiro, Kaio Moraes Farias, Durvanei Augusto Maria, Giselle Cerchiaro, Vani Xavier Oliveira

**Affiliations:** † Laboratory of Viral Biotechnology, 196591 Butantan Institute, São Paulo 05508-210, Brazil; ‡ Paulista School of Medicine, Postgraduate Program in Molecular Biology, Federal University of São Paulo, São Paulo 04044-020, Brazil; § Faculty of Medicine, University of São Paulo, São Paulo 05508-220, Brazil; ∥ Laboratory of Development and Innovation, 28133 Butantan Institute, São Paulo 05585-000, Brazil; ⊥ Center for Natural and Human Sciences, 74362 Federal University of ABC, Santo André 09280-560, Brazil; # Biochemistry Department, University Center FMABC, Santo André 09060-650, Brazil

**Keywords:** IsCT1, Host Defense Peptides, Anticancer Peptide, Photodynamic Therapy, Synergy, Immune-Associated Activity, Scorpion Venom Peptides

## Abstract

Host Defense Peptides (HDPs) are key components of the innate immune system and promising candidates for anticancer therapy due to their ability to interact with cell membranes. In this study, we investigated the structure–activity relationship of the scorpion venom-derived HDP IsCT1 and its rationally designed analogs against breast cancer models. The analog AKFK-IsCT1 emerged as the lead compound, exhibiting potent antitumor activity while reducing cytotoxicity toward nontumorigenic cells. Notably, AKFK-IsCT1 maintained an optimal balance of positive charge and helical conformation, which proved more critical for anticancer efficacy than charge alone. When combined with photodynamic therapy using hypericin, AKFK-IsCT1 displayed remarkable synergistic effects, enabling substantial dose reduction of both agents. In a triple-negative breast cancer mouse model, AKFK-IsCT1 treatment reduced tumor burden and elicited immune-associated responses, supporting its potential as a selective and multifunctional therapeutic strategy against aggressive breast cancers.

## Graphical abstract

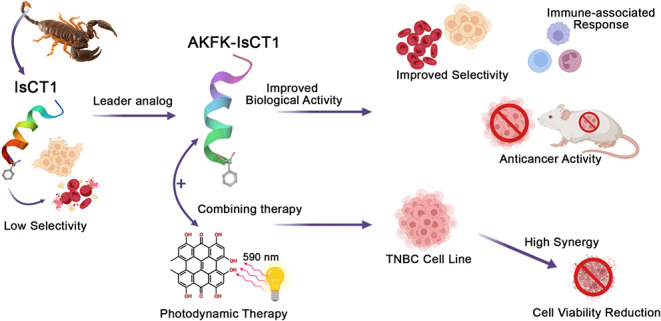

Breast cancer has a high global prevalence, with 2.3 million new cases in 2020,^1^ representing approximately one in every eight cancer diagnoses. It is also a leading cause of mortality among women, accounting for about 685,000 deaths in the same year.^2,3^ The main breast cancer subtypes are hormone receptor-positive/ERBB2 negative (HR^+^/ERBB2^–^), ERBB2 positive (ERBB2^+^), and triple-negative (TNBC).^4^ TNBC is an aggressive and highly invasive subtype, responsible for approximately 15% of all breast cancer cases. It is associated with higher mortality than other subtypes, with nearly 40% of deaths occurring in the first year after diagnosis and a postoperative recurrence rate of approximately 25%.^5^

Current breast cancer treatments include surgery, radiotherapy, monoclonal antibodies, endocrine therapy, and chemotherapy.^4^ However, treatment efficacy is often limited by various cellular resistance mechanisms.^6^ Consequently, the search for new molecules and therapeutic strategies against breast cancer—particularly TNBC—is critical. Host Defense Peptides (HDPs) represent a promising candidate in this context. HDPs are short-length, amphipathic peptides, usually cationic, that typically adopt α-helical structure.^7^ They exhibit a broad spectrum of biological activities against bacteria,^8–10^ yeast,^11,12^ parasites,^13–15^ viruses,^16,17^ and tumor cells,^14,18^ and may also exert immunomodulatory effects.^19,20^ Additionally, HDPs can act synergistically or complementarily with other cancer therapies, enabling dose reduction of the agents involved, as demonstrated by synergistic interaction between Gramicidin A and Doxorubicin in colorectal cancer cells.^21^ When combined with photodynamic therapy (PDT), many HDPs display additive or synergistic effects, achieving biological activity at reduced concentrations.^22^ Using porphyrin as a photosensitizer, Buforin II, Magainin 2, and Apidaecin synergized with PDT to induce complete loss of A549 lung cancer cell viability within 24 h.^23^

IsCT1 (ILGKIWEGIKSLF-NH_2_) is a cationic α-helical HDP naturally found in the venom of the scorpion *Opisthacanthus madagascariensis*. It exhibits pronounced biological activity against Gram-positive and Gram-negative bacteria, as well as yeast, and moderate hemolytic activity.^12,24^ Its positive net charge at physiological pH arises from the presence of two lysine residues, the protonated N-terminus, and C-terminal amidation, the latter eliminating a potential negative charge contribution and thereby reinforcing electrostatic interactions with negatively charged biological membranes.^25^

In a previous study, we synthesized a series of synthetic IsCT1 analogs containing one to four amino acid substitutions in either the hydrophobic or hydrophilic region of its amphipathic helical structure (Table 1). These peptides were characterized and evaluated for their antimicrobial activity against Gram-positive and Gram-negative bacteria, as well as for their hemolytic effects on human erythrocytes. That work identified a lead analog containing four substitutions and a +4 net charge, which exhibited enhanced antimicrobial activity and reduced hemolysis.^26^

**Table 1:** Peptide Sequence, Molecular Weight, Purity, Helical Fraction, and Net charge^26^

			^a^Helical fraction		
Peptide	Sequence	Purity (%)	Water	TFE	*z* ^b^
IsCT1	ILGKIWEGIKSLF-NH_2_	95	0.07	0.45	+2
P-IsCT1	ILGKFW**P**GIKSLF-NH_2_	95	0.08	0.18	+3
KP-IsCT1	IL**K**KIWE**P**IKSLF-NH_2_	99	0.04	0.14	+3
AKFK-IsCT1	**A**L**K**K**F**WE**K**IKSLF-NH_2_	96	0.00	0.38	+4
AFPK-IsCT1	**A**LGK**F**W**PK**IKSLF-NH_2_	98	0.06	0.15	+4
KKK-IsCT1	IL**K**KIW**K**G**K**KSLF-NH_2_	99	0.05	0.17	+6
KKPK-IsCT1	IL**K**KIW**KPK**KSLF-NH_2_	99	0.07	0.10	+6

a Helical fraction obtained from Oliveira et al., 2021.^26^

b Net charge.

In the present study, we investigate the anticancer activity on breast cancer models of the same series of synthetic IsCT1 analogs and assess their potential synergistic interactions with photodynamic therapy.

## Results and Discussion

### Peptide Design, Synthesis, and Structural Characterization

In a previous work, we synthesized IsCT1, an α-helical HDP, and six analogs designed with 1 to 4 amino acid substitutions and verified their antimicrobial and hemolytic activities^26^ (Table 1).

In general, helical HDPs exhibit high biological activity, and several exhibit anticancer activity.^14,18,20,27–29^ The anticancer activity of these helical HDPs is mainly due to their physicochemical characteristics, such as positive net charge, hydrophobicity, and amphipathicity.^9,30^ The ability of amphipathic α-helical peptides to present their hydrophobic amino acid residues on one face of the helix and the hydrophilic amino acid residues on the opposite face may promote greater interaction between the peptide and the negative charges present in the cancer cell membrane, such as sialic acid, anionic phospholipids, and *O*-glycosylated mucins.^31,32^ Some previous works reported modifications to the biological activity of IsCT1, such as reduced hemolytic activity through the insertion of proline at position 8 or the replacement of a negative residue (glutamic acid),^33,34^ and enhanced antimicrobial activity by adding lysine to increase the net charge.^12,35,36^

The IsCT1 analogs differ from the native peptide by amino acid substitutions located on the hydrophobic and hydrophilic faces of the predicted amphipathic α-helical structure, primarily at positions 1, 3, 5, 7, 8, and 9. As a consequence of these substitutions, the peptides display net positive charges ranging from +2 to +6 (Table 1). Peptides were obtained by solid-phase peptide synthesis with the Fmoc strategy and purified by RP-HPLC, achieving final purity ≥95% (Table 1 and Figure S1). The helical structure and physicochemical parameters, such as hydrophobicity (H), hydrophobic moment (μH), and polar–nonpolar residues proportion (P/N) for each peptide were obtained using the online server Heliquest^37^ (Figure 1).

**Figure 1:**
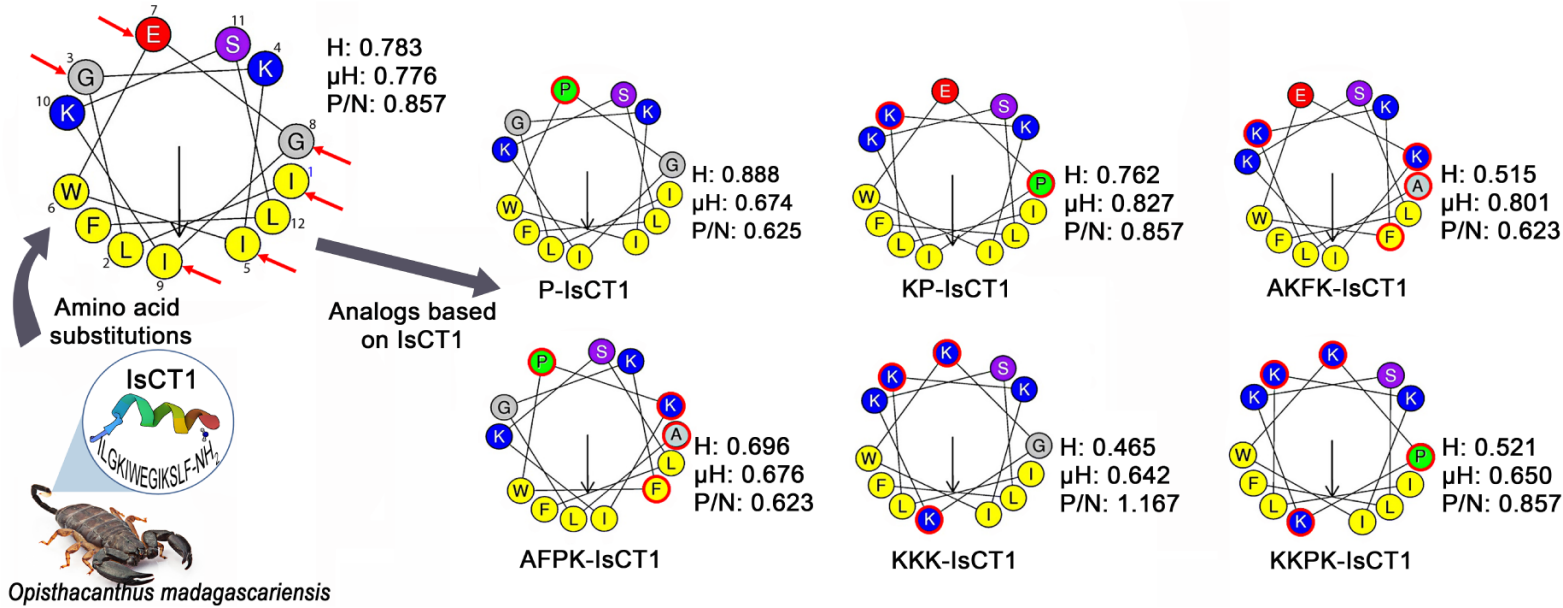
Helical wheel projections of IsCT1 and its analogs. Circle colors indicate amino acid classes: yellow, aromatic and aliphatic hydrophobic residues; gray, residues with near-neutral hydrophobicity; blue, basic positively charged residues; purple, polar uncharged residues; red, negatively charged residues; green, the cyclic amino acid proline. Black arrows showing the direction and magnitude of the hydrophobic moment, calculated by the Heliquest online server.^37^ Red arrows and circles highlight the positions along the peptide sequence where substitutions were introduced. Adapted with permission from reference 26. Copyright © 2021, American Chemical Society.

The secondary structure of the synthetic peptides used in this study had been previously characterized by Oliveira et al.^26^ through circular dichroism (CD) spectroscopy under different solvent conditions. That earlier analysis showed that specific amino acid substitutions altered the helical propensity of the analogs relative to native IsCT1 (Table 1). As expected, replacement with proline, an amino acid known to introduce backbone distortions and increase local flexibility,^35^ led to reduced helicity, even in TFE, a medium that typically stabilizes α-helical structures. The KKK-IsCT1 variant displayed diminished helical content associated with an elevated net charge and a higher proportion of polar residues (P/N > 1; Figure 1), which is consistent with decreased hydrophobicity and impaired amphipathic organization.^9^ Meanwhile, the AKFK-IsCT1 analog retained a helical profile similar to the native peptide while increasing the net charge to +4; notable features of this analog, including its reduced cytotoxicity toward nontumorigenic cells and anticancer performance, are discussed below.

### Structure–Activity Studies and Biological Assays

The biological activity of IsCT1-based peptides was directly associated with the integrity of the peptide scaffold. As previously reported, disruption of the helical structure lead to reduced hemolytic and antimicrobial activity.^26^ In the present study, a similar trend was observed for anticancer activity, as loss of helicity also resulted in diminished cytotoxicity against breast cancer cells (Table 2). P-IsCT1 exhibited a marked reduction in activity, whereas the analogs KP-IsCT, AFPK-IsCT1, KKK-IsCT1, and KKPK-IsCT1 were inactive at the tested concentrations in all evaluated cell lines.

**Table 2:** Half-Maximal Inhibitory Concentration (IC_50_, μM) of IsCt1 and Its Analogs against Non-Tumorigenic (MCF-10A) and Breast Cancer Cell Lines (MCF-7, MDA-MB-231, and 4T1), and Corresponding Therapeutic Index (TI)^a^

	IC_50_ ± SD (μM) and Therapeutic Index						
Peptide	MCF-10A	MCF-7	TI	MDA-MB-231	TI	4T1	TI
IsCT1	63 ± 12	76 ± 13	0.8	28 ± 5*	2.2	29 ± 5*	2.1
P-IsCT1	122 ± 10	99 ± 9	1.2	74 ± 9*	1.6	NT	NA
KP-IsCT1	>128	>128	NA	>128	NA	NT	NA
AKFK-IsCT1	106 ± 6	65 ± 10*	1.6	47 ± 11*	2.2	24 ± 10*	4.4
AFPK-IsCT1	>128	>128	NA	>128	NA	NT	NA
KKK-IsCT1	>128	>128	NA	>128	NA	NT	NA
KKPK-IsCT1	>128	>128	NA	>128	NA	NT	NA

a NA: not available—it was not possible to calculate because the IC_50_ value was not reached; TI: calculated as the ratio of the IC50 for MCF-10A to that for the other cell lines; NT: not tested—only leader peptides were tested at the 4T1 cell line. IC_50_ values were obtained using the AAT Bioquest online server (aatbio.com/tools/ic50-calculator). * *p* < 0.00006.

VmCT, another scorpion venom-derived peptide, shares structural and sequence similarities with IsCT1. Previous studies have shown that VmCT analogs display enhanced biological activity correlated with increased net positive charge.^14,38^ However, this relationship was not observed for IsCT1 analogs. Notably, KKK-IsCT1 and KKPK-IsCT1, both carrying a net charge of +6, failed to exhibit activity in the tested cell lines, indicating that increased charge alone is insufficient to enhance the anticancer activity of IsCT1 derivatives.

In turn, the analog AKFK-IsCT1 exhibited a net charge of +4 and helical content in membrane-mimetic environments, such as TFE,^39^ comparable to the native peptide (Table 1). Despite displaying reduced global hydrophobicity relative to IsCT1, AKFK-IsCT1 maintained a high hydrophobic moment (Figure 1) and a pronounced helix-forming propensity (Table 1), supporting the hypothesis that a stable amphipathic α-helix may form upon membrane contact.^40^ This optimized physicochemical balance distinguishes AKFK-IsCT1 as a lead analog, consistent with its previously reported enhanced antimicrobial activity *in vitro* and *in vivo* ^26^ and its promising anticancer activity in breast cancer cell lines at concentrations that did not induce cytotoxicity in nontumorigenic mammary epithelial cells (Table 2). In contrast, the AFPK-IsCT1 analog, although presenting a similar net charge, exhibited reduced helicity and a lower hydrophobic moment, likely due to helix-disrupting residues, which correlates with its diminished biological activity.^39,40^ These findings reinforce that α-helical structuring and hydrophobic patterning, rather than increased charge alone, are critical determinants of therapeutic efficacy.^40,41^ Indeed, helical conformation of HDPs plays a central role in antitumor activity by promoting selective interactions with the negatively charged and structurally disordered compared membrane of cancer cells and enabling multiple mechanisms of action, including membrane depolarization, membrane destabilization via pore formation (barrel-stave or toroidal pores), carpet-like lytic effects, and cellular internalization, leading to intracellular targeting.^42–44^

As shown in Table 2, both IsCT1 and its analog AKFK-IsCT1 were more active against TNBC cell lines (MDA-MB-231 and 4T1) than against the HR^+^ MCF-7 cells. While IsCT1 displayed higher potency overall, AKFK-IsCT1 maintained relevant antitumor activity against breast cancer cell lines and exhibited reduced cytotoxicity toward nontumorigenic MCF-10A cells (IC_50_ ≈ 100 μM) compared with the native peptide (IC_50_ ≈ 60 μM). These results indicate that sequence modification in AKFK-IsCT1 preserves anticancer activity while attenuating cytotoxic effects on nontumorigenic cells, supporting its improved safety profile relative to IsCT1.

### Synergy between Peptides and Photodynamic Therapy

Although IsCT1 and its analog AKFK-IsCT1 exhibited measurable anticancer activity, both peptides showed relatively low therapeutic index (TI) values when applied as monotherapy (Table 2), indicating a limited safety margin between antitumor efficacy and cytotoxicity toward nontumorigenic cells.^45^ In this context, combination strategies were investigated as an approach to reduce effective concentrations while minimizing off-target toxicity.^46^

Photodynamic therapy using hypericin—a secondary metabolite of *Hypericum perforatum*L.—(PDT-Hyp) was selected due to its well-established ability to generate reactive oxygen species (ROS), particularly singlet oxygen, upon photoactivation in the 590–600 nm range, leading to oxidative cellular damage and loss of viability.^47,48^

When applied individually, PDT-Hyp displayed minimal cytotoxicity toward nontumorigenic MCF-10A cells at concentrations up to 1.0 μM, whereas both breast cancer cell lines showed a concentration-dependent reduction in viability, with significant effects observed from 0.5 μM hypericin onward (Figure 2). Similarly, IsCT1 and AKFK-IsCT1 alone produced limited cytotoxic effects at concentrations below 4.0 μM across all tested cell lines (Figure 3), establishing an appropriate baseline for evaluating combination effects.

**Figure 2:**
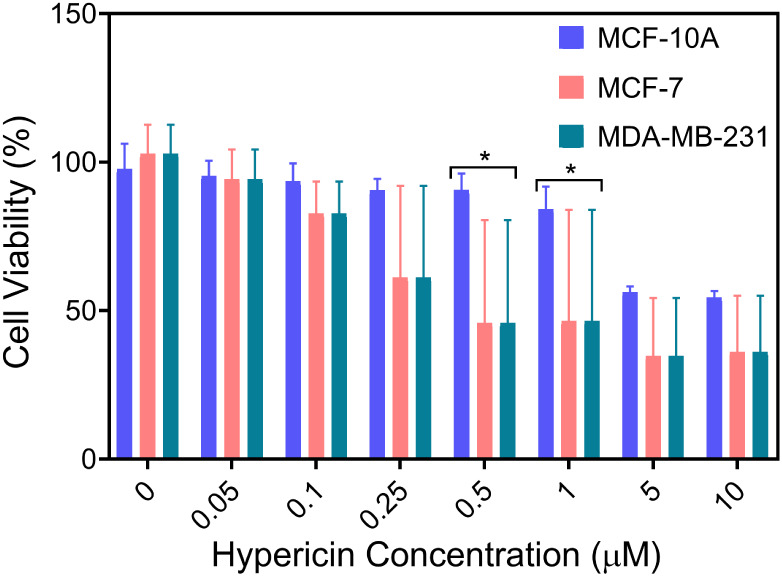
Treatment of breast cell lines with different concentrations of Hypericin. Cells were treated with 10 min of internalization and irradiation at 590 nm for 10 min, followed by incubation at 37 °C and 5% CO_2_, in the dark for 4 h. Blue bars represent MCF-10A, orange bars represent MCF-7, and green bars represent MDA-MB-231. The experiment was performed in three independent replicates. Statistical significance was determined using two-way ANOVA followed by the Tukey test, *p < 0.02.

**Figure 3:**
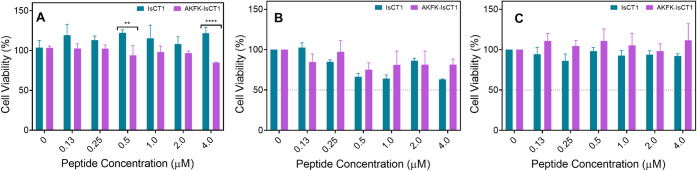
Cell viability of the cell lines MCF-10A (A), MCF-7 (B), and MDA-MB-231 (C) after treatments with IsCT1 (green bars) or AKFK-IsCT1 (purple bars) at concentrations up to 4.0 μM, followed by incubation at 37 °C and 5% CO_2_ for 4 h. Green bars represent IsCT1, and pink bars represent AKFK-IsCT1. Data are expressed as mean ± SD of three independent experiments performed in triplicate. Statistical significance was determined using two-way ANOVA followed by Tukey test, **p < 0.005; ****p < 0.0001.

Based on the results obtained from cell treatments with hypericin or peptides, two treatment protocols were designed to evaluate the combined effects of hypericin-mediated photodynamic therapy (PDT-Hyp) with the peptides: Protocol 1, in which PDT-Hyp was applied prior to peptide exposure, and Protocol 2, in which peptide treatment preceded PDT-Hyp. In both protocols, noncytotoxic hypericin concentrations (0.05 or 0.1 μM) were combined with peptide concentrations ranging from 0 to 4.0 μM and tested in breast cancer cell lines (MCF-7 and MDA-MB-231) as well as in the nontumorigenic epithelial breast cell line MCF-10A.

The combined treatment revealed a strong dependence on treatment sequence. Overall, Protocol 1 consistently resulted in a greater reduction in cell viability compared with Protocol 2 across all cell lines and peptide concentrations (Figure 4A–C; Figure S2). Under Protocol 1, the association of PDT-Hyp (0.05 μM) with AKFK-IsCT1 induced a pronounced reduction in viability in MCF-7 and MDA-MB-231 cells, while exerting minimal effects on MCF-10A cells. Increasing the hypericin concentration to 0.1 μM further enhanced anticancer activity without proportionally increasing cytotoxicity in nontumorigenic cells (Figure S2), indicating a favorable activity profile for AKFK-IsCT1 under this protocol. In contrast, combinations involving IsCT1, although effective against tumor cells, were associated with mild cytotoxic effects in MCF-10A cells, particularly at higher peptide concentrations and at Hyp 0.1 μM, suggesting reduced selectivity.

**Figure 4:**
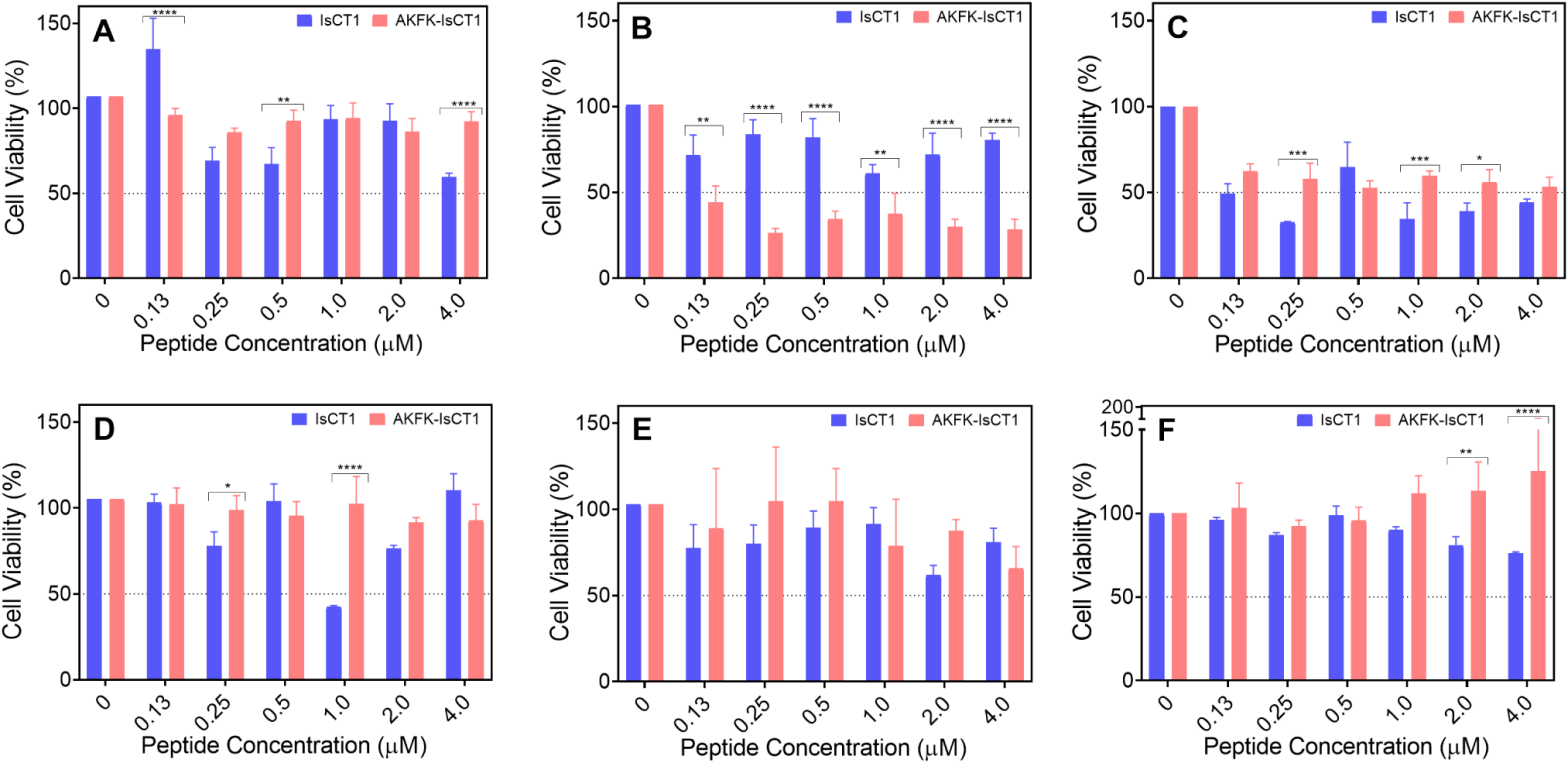
Cell viability after combined treatment with photodynamic therapy using hypericin (PDT-Hyp, 0.05 μM) and peptides under two treatment protocols. Protocol 1 (PDT-Hyp followed by peptide exposure) was applied to (A) MCF-10A, (B) MCF-7, and (C) MDA-MB-231 cells, whereas Protocol 2 (peptide exposure followed by PDT-Hyp) was applied to (D) MCF-10A, (E) MCF-7, and (F) MDA-MB-231 cells. Blue bars represent IsCT1, and red bars represent AKFK-IsCT1. Peptides were tested at concentrations ranging from 0.01 to 4.0 μM. Cells were incubated at 37 °C and 5% CO_2_ for 4 h following treatment. Data are expressed as mean ± SD of three independent experiments performed in triplicate. Statistical significance was determined using two-way ANOVA followed by Tukey test, *p < 0.05; **p < 0.005; ***p < 0.0005; ****p < 0.0001.

Protocol 2 resulted in limited or inconsistent reductions in cell viability regardless of peptide identity or hypericin concentration (Figure 4D–F; Figure S2), indicating that peptide pretreatment does not potentiate PDT-Hyp efficacy to the same extent as Protocol 1.

Pharmacological interaction analysis using SynergyFinder 3.0 under the Bliss independence model^49^ corroborated these observations (Figure 5; Figure S3). For IsCT1, synergistic interactions were consistently observed across all cell lines and both protocols, indicating a lack of selectivity toward malignant cells. In contrast, AKFK-IsCT1 displayed a protocol- and cell-dependent interaction profile (Table 3; Table S1). Under Protocol 1, strong synergistic interactions were observed in MCF-7 and MDA-MB-231 cells, whereas predominantly additive effects were detected in MCF-10A cells. Under Protocol 2, synergy was reduced or lost, culminating in an antagonistic interaction in MDA-MB-231 cells. Analyses using the Loewe additivity and Highest Single Agent (HSA) models yielded results consistent with the Bliss data (Table S1).

**Figure 5:**
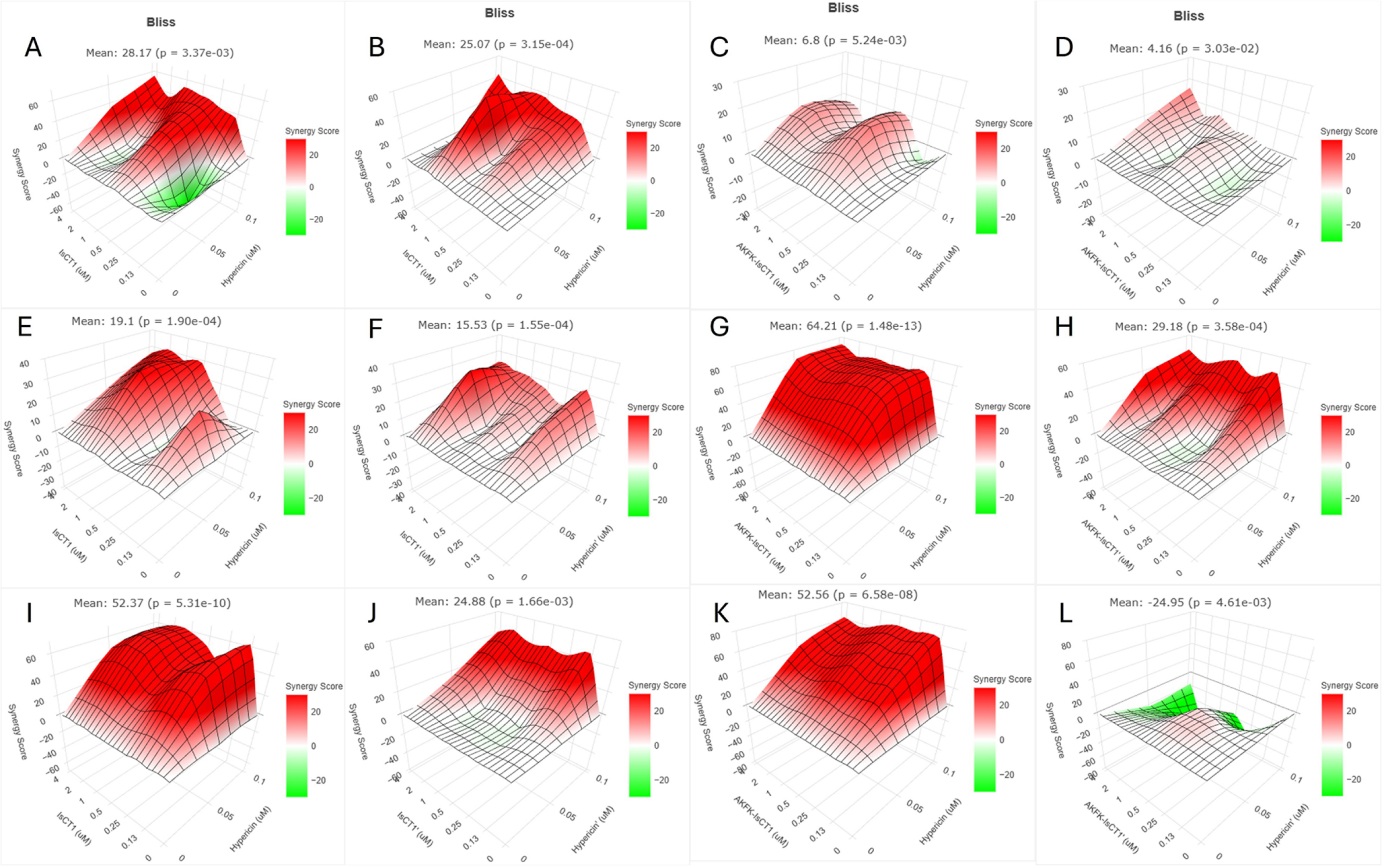
Three-dimensional Bliss synergy response surfaces for the combined treatment of PDT-Hyp and peptides applied under different treatment protocols. Protocol 1 was evaluated with IsCT1 (A, E, I) or AKFK-IsCT1 (C, G, K), whereas Protocol 2 was evaluated with IsCT1 (B, F, J) or AKFK-IsCT1 (D, H, L). Panels in the first row (A–D) correspond to MCF-10A cells, the second row (E–H) to MCF-7 cells, and the third row (I–L) to MDA-MB-231 cells. Color coding represents Bliss synergy scores, where green indicates antagonism (≤0), intermediate colors between white and light red indicate additive effects (>0 and ≤10), and red indicates synergistic interactions (>10). Color intensity is proportional to the magnitude of the interaction score. Bliss synergy scores and response surfaces were generated from complete dose–response matrices using SynergyFinder 3.0.

**Table 3:** Pharmacological Effect of Different Protocols Combining PDT-Hyp with IsCt1 or AKFK-IsCt1 Was Applied to Different Cell Lines, Using Bliss Method

Cell line	Combination^a^	Protocol	Bliss Synergy Score	Effect
MCF-10A	PDT-Hyp + IsCT1	1	28.17	Synergy
	IsCT1 + PDT-Hyp	2	25.07	Synergy
	PDT-Hyp + AKFK-IsCT1	1	6.8	Additive
	AKFK-IsCT1 + PDT-Hyp	2	4.16	Additive
MCF-7	PDT-Hyp + IsCT1	1	19.1	Synergy
	IsCT1 + PDT-Hyp	2	15.53	Synergy
	PDT-Hyp + AKFK-IsCT1	1	64.21	Synergy
	AKFK-IsCT1 + PDT-Hyp	2	29.19	Synergy
MDA-MB-231	PDT-Hyp + IsCT1	1	52.37	Synergy
	IsCT1 + PDT-Hyp	2	24.88	Synergy
	PDT-Hyp + AKFK-IsCT1	1	52.56	Synergy
	AKFK-IsCT1 + PDT-Hyp	2	–24.95	Antagonist

a Express the treatment application order, equivalent to protocol 1 or 2.

Mechanistically, these protocol-dependent effects can be explained by the interplay between hypericin internalization and peptide-induced membrane perturbation. Hypericin is a highly lipophilic photosensitizer that incorporates into cellular membranes by passive diffusion and accumulates in intracellular membranous compartments, including the cytoplasmic leaflet of the plasma membrane, endoplasmic reticulum, and Golgi apparatus.^50,51^ Its uptake, aggregation state, and photodynamic efficiency are strongly influenced by membrane integrity, lipid organization, and membrane potential. Early membrane perturbations have been shown to impair hypericin internalization and alter its subcellular distribution, resulting in reduced photodynamic efficacy.^52,53^

Cationic α-helical peptides such as IsCT1 and AKFK-IsCT1 interact with negatively charged membrane components, inducing membrane destabilization through carpet-like mechanisms or toroidal pore formation.^54–56^ In addition to structural disruption, these peptides are capable of inducing membrane depolarization. Previous studies demonstrated that peptides related to IsCT1 promote rapid, concentration-dependent membrane depolarization, with AKFK-IsCT1 exhibiting a greater depolarizing capacity than the native peptide.^26^ This enhanced ability to dose-dependent collapse membrane potential provides a mechanistic basis for the observed differences between protocols.

When AKFK-IsCT1 is applied after hypericin internalization (Protocol 1), peptide-induced membrane depolarization and controlled destabilization likely occur after the photosensitizer has reached its intracellular targets, thereby potentiating ROS-mediated phototoxicity while preserving selectivity. Conversely, peptide pretreatment (Protocol 2) may induce early membrane depolarization and structural perturbation, hindering hypericin uptake and intracellular redistribution,^52,57^ which is consistent with the reduced or antagonistic interactions observed, particularly in MDA-MB-231 cells.

Taken together, these results indicate that both peptide sequence and treatment order influence the outcome of PDT–peptide combinations. Notably, when PDT-Hyp was applied at a reduced concentration (0.05 μM) followed by AKFK-IsCT1 (Protocol 1), antitumor activity was achieved at substantially lower peptide concentrations than those required in monotherapy. Under these conditions, AKFK-IsCT1 displayed measurable activity at submicromolar levels (≈0.13 μM), whereas concentrations in the micromolar range were necessary to elicit comparable effects when the peptide was applied alone. Importantly, this enhanced activity was obtained without a proportional increase in cytotoxicity toward nontumorigenic MCF-10A cells, resulting in a more favorable selectivity profile. These findings suggest that the combined protocol enables effective biological responses using reduced doses of both the photosensitizer and the peptide, thereby broadening the apparent therapeutic window. Overall, the data support the relevance of dose reduction and treatment sequence as key parameters in optimizing PDT–peptide combinations, while avoiding definitive conclusions regarding the underlying mechanisms.

### Inhibition of Tumor Growth in Mice

IsCT1 and its analog AKFK-IsCT1 showed preferential activity against the TNBC cell line, consistently observed across all in vitro assays (Table 2). The mouse 4T1 breast tumor model is an aggressive cancer model, corresponding to TNBC in vitro models, highly tumorigenic and invasive, and capable of spontaneous metastasis from the primary mammary tumor to distant sites such as lymph nodes, blood, liver, lung, brain, and bone. The 4T1 tumor has several characteristics that make it a suitable model for the study of human mammary cancer using animal models.^58^

Animals were inoculated with the 4T1 cell line and, 7 days after, were treated with intratumoral injections of peptides (groups IsCT1 or AKFK-IsCT1) or saline solution (control group). No significant weight differences were observed between treated and untreated groups during the experimental protocol, indicating nontoxicity (Figure 6).

**Figure 6:**
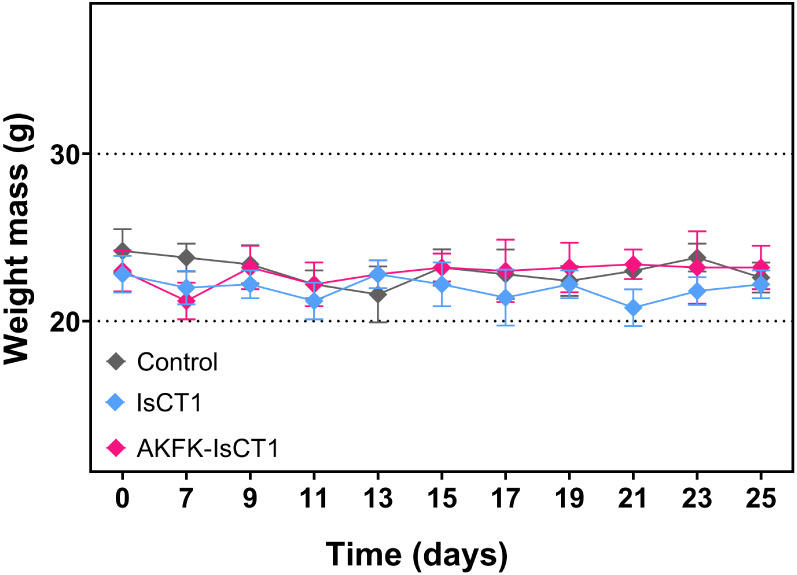
Toxicity of peptides was assessed through animal weight monitoring during the experimental protocol. Mean values ± standard deviations, and statistical differences were obtained by ANOVA, followed by Tukey’s multiple comparison test.

The *in vivo* antitumor effects observed following treatment with IsCT1 and AKFK-IsCT1 were supported by complementary volumetric, gravimetric, histopathological, hematological, and functional analyses. Although tumor volume increased over time in both control and treated groups (Figure 7A), a marked and statistically significant reduction in final tumor mass was observed in animals treated with either peptide (Figure 7B). This apparent discrepancy between tumor volume and mass is likely attributable to the presence of peritumoral fluid or edema, which may artificially inflate volumetric measurements without reflecting viable tumor burden, as previously reported for aggressive breast cancer models such as 4T1.^59,60^ Therefore, tumor mass measurements provide a more reliable indicator of treatment efficacy in this model.

**Figure 7:**
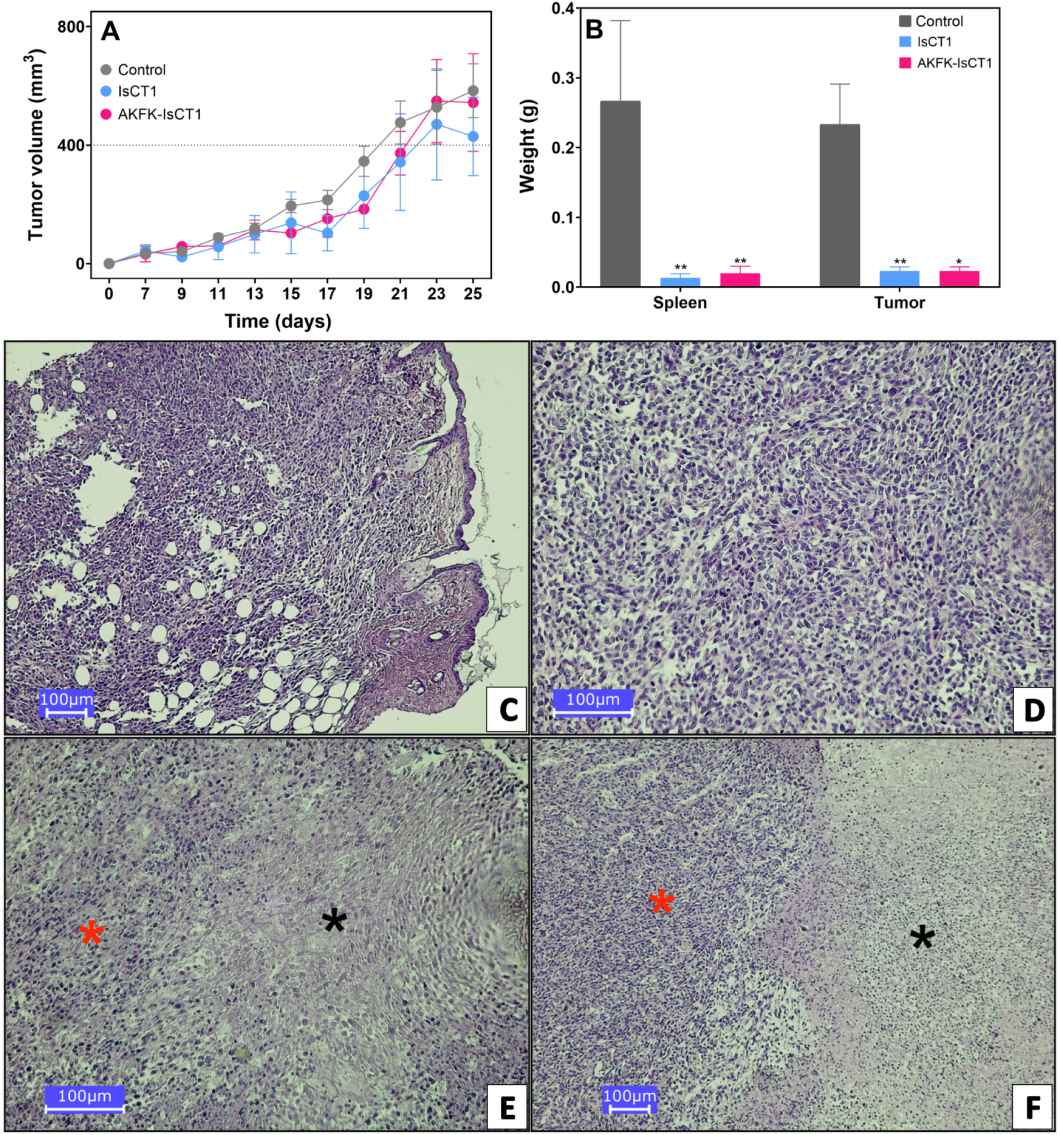
(A) Tumor volume of the control, IsCT1, and AKFK-IsCT1 groups obtained throughout the experimental protocol. (B) Final values of spleen and tumor mass in the control and treated groups. Organs were collected at the end of the experimental period and immediately weighed. The groups were indicated as control in gray, IsCT1 in blue, and AKFK-IsCT1 in pink. Mean values ± standard deviation and statistical differences were obtained by ANOVA followed by Tukey’s multiple comparison test; *p < 0.03; **p < 0.001. Images C–F present a histopathological analysis of mammary tumors. C and D indicate the control group and show an expansive, densely cellular, nonencapsulated, and reasonably well-demarcated neoplastic mass. E (IsCT1) and F (AKFK-IsCT1) groups show a large area of coagulative and lytic tumor necrosis. Within and around the tumor, infiltrates of neutrophils, lymphocytes, plasma cells, and macrophages are present. Note the areas of intense necrosis (asterisks) and areas of adjacent tumor tissue (red asterisk). (H&E 10× and 20×).

Histopathological examination of mammary tumors further corroborated the antitumor activity of both peptides. Tumors from control animals displayed densely cellular, expansile, nonencapsulated neoplastic masses, consistent with an aggressive and highly proliferative phenotype. In contrast, tumors from IsCT1- and AKFK-IsCT1-treated animals exhibited extensive areas of coagulative and lytic necrosis, together with well-demarcated transitions between viable and necrotic tissue (Figure 7C–F). The presence of infiltrating immune cells, including neutrophils, lymphocytes, and macrophages, within and around necrotic regions suggests activation of local inflammatory responses commonly associated with effective tumor cell death *in vivo*.^58,61–63^

Systemic effects were further evaluated through hematological analyses (Table S2). As expected for the 4T1 model, control animals developed pronounced leukocytosis and splenomegaly, features often linked to tumor-driven inflammatory and metastatic processes.^64^ In contrast, animals treated with IsCT1 or AKFK-IsCT1 did not show comparable spleen enlargement, despite elevated circulating leukocyte counts. Notably, AKFK-IsCT1-treated animals exhibited increased lymphocyte and monocyte counts relative to controls, a pattern that may reflect modulation of immune cell dynamics rather than nonspecific toxicity. Importantly, these changes occurred in the absence of overt alterations in organ mass or histological evidence of systemic damage, supporting a preserved physiological balance.

Functional assessment of immune and tumor-derived cells *ex vivo* provided additional insight into the biological relevance of these observations. Tumor cells isolated from animals treated with either peptide displayed significantly reduced proliferative capacity, even when stimulated with the mitogen concanavalin A (Figure 8A). This sustained reduction in proliferation suggests that *in vivo* peptide treatment induced lasting alterations in tumor cell viability or cell-cycle competence, consistent with effective antitumor pressure.^63,65^ In contrast, spleen-derived cells from treated animals retained normal proliferative responses under basal conditions, mitogenic stimulation, and peptide re-exposure (Figure 8B). Preserved responsiveness to concanavalin A, a classical T-lymphocyte mitogen, indicates that systemic peptide treatment did not impair splenic immune cell function.^66,67^

**Figure 8:**
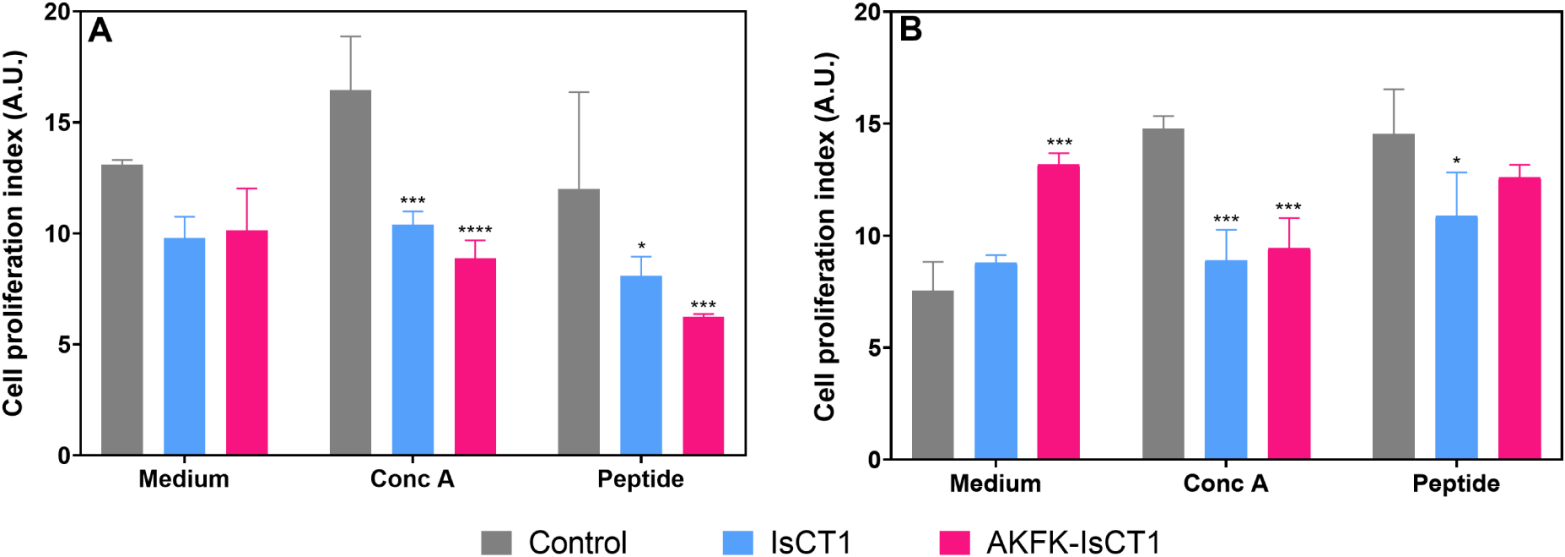
Cell proliferation index of cells isolated from (A) mammary tumors and (B) spleens of animals in the Control, IsCT1, and AKFK-IsCT1 groups. Cells were labeled with CFSE and cultured under three conditions: medium alone (Medium), medium with concanavalin A (Con A), or medium containing the same peptide given to the donor animals (Peptide). Gray bars depict the untreated group (control), blue bars show the IsCT1-treated group, and pink bars illustrate the AKFK-IsCT1-treated group. Data are presented as mean ± SD of three independent experiments. Statistical significance was assessed by ANOVA followed by Tukey’s post hoc test (*p < 0.01; **p < 0.001; ***p < 0.0001).

Taken together, these integrated *in vivo* and *ex vivo* findings indicate that IsCT1 and AKFK-IsCT1 reduce tumor burden and tumor cell proliferative capacity while largely preserving immune organ integrity and splenic cell function. The differential effects observed between tumor and immune compartments support a degree of biological selectivity, rather than generalized cytotoxicity, and are consistent with previously reported profiles of host-defense peptides with anticancer activity.^43,56,68^ This balanced activity profile strengthens the translational relevance of these peptides as candidates for further investigation into anticancer strategies.

## Conclusion

Host defense peptides (HDPs) represent a promising class of molecules for the development of novel anticancer therapeutics strategies, as they can combine direct effects on tumor cells with immunomodulatory properties, often at concentrations associated with limited toxicity to nonmalignant tissues.^19,69,70^ Among these peptides, IsCT1, an α-helical HDP originally isolated from scorpion venom, has attracted interest due to its broad spectrum of biological activities. However, despite its moderate antitumor effects, the relatively high hemolytic activity of IsCT1 underscores the importance of peptide optimization to improve its therapeutic profile.

In previous work, the IsCT1-derived analog AKFK-IsCT1 was designed to reduce hemolytic activity while preserving biological function and was shown to display robust antibacterial activity both *in vitro* and *in vivo*.^26^ Building on these findings, the present study demonstrates that AKFK-IsCT1 also exhibits relevant antitumor potential. The results indicate that subtle sequence modifications, including a moderate increase in net positive charge while maintaining α-helical structure, can favorably modulate the balance between efficacy and selectivity in IsCT1-based peptides.

AKFK-IsCT1 showed consistent activity against breast cancer cell lines, with a more pronounced effect on aggressive phenotypes, such as MDA-MB-231 and 4T1, while exerting minimal cytotoxicity toward the nontumorigenic MCF-10A cell line. Notably, when combined with photodynamic therapy using hypericin (PDT-Hyp), AKFK-IsCT1 displayed marked synergistic interactions, enabling antitumor activity at peptide concentrations approximately 400-fold lower than those required for monotherapy, as well as at reduced photosensitizer doses. These findings support the potential of this combined strategy to enhance therapeutic efficacy while limiting exposure to higher drug concentrations.

Furthermore, *in vivo* experiments have shown that AKFK-IsCT1 treatment can reduce tumor burden in the murine breast cancer model. Histopathological analyses revealed extensive areas of tumor necrosis, accompanied by changes in hematological parameters suggestive of immune system engagement. Although the precise mechanisms underlying these effects require further investigation, the observed combination of direct antitumor activity and modulation of host immune responses suggests that AKFK-IsCT1 may contribute to an antitumor microenvironment conducive to tumor control.

Taken together, this study supports the notion that rationally optimized HDPs, such as AKFK-IsCT1, particularly when integrated with complementary modalities like photodynamic therapy, represent a promising avenue for the development of alternative therapeutic approaches for solid tumors, including triple-negative breast cancer. Further studies aimed at elucidating the mechanistic basis of these effects and evaluating long-term safety will be essential to advance this strategy toward translational applications.

## Materials and Methods

### Solid-Phase Peptide Synthesis, Purification, and Analysis

The synthetic IsCT1 peptide and its rationally designed analogs used in the present study were previously developed and characterized by Oliveira et al.^26^ As described in that earlier work, the peptides were synthesized using solid-phase peptide synthesis with the fluorenylmethyloxycarbonyl (Fmoc) strategy on Rink Amide MBHA resin (AAPPTec LLC, Louisville, Kentucky). The crude products were subsequently lyophilized, purified by semipreparative reverse-phase high-performance liquid chromatography (RP-HPLC), and fully characterized by liquid chromatography–electrospray ionization mass spectrometry (LC/ESI-MS), and circular dichroism (CD).

### Cell Lines and Culture Conditions

The triple-negative breast cancer (TNBC) cell lines human MDA-MB-231 (CRM-HTB-26) and murine 4T1 (CRL-2539), human adenocarcinoma cell line MCF-7 (HTB-22), and the human breast epithelial cell line MCF-10A (CRL-10317) were obtained from American Type Culture Collection (ATCC). The MDA-MB-231, 4T1, and MCF-7 cell lines were maintained at 37 °C in 5% CO_2_, using Dulbecco’s Modified Eagle Medium (DMEM) supplemented with 10% inactivated fetal bovine serum and 1% penicillin-streptomycin. Additionally, MCF-7 medium was supplemented with human recombinant insulin (10 mg L^–1^). The MCF-10A cell line was maintained in DMEM/F-12 (DMEM/nutrient mixture F-12 Ham) supplemented with 5% inactivated horse serum, 100 ng mL^–1^ cholera toxin, human recombinant insulin (10 mg L^–1^), and 1% penicillin-streptomycin, at 37 °C in 5% CO_2_.

### Cell Viability Assay

Cell viability was assessed using the MTT assay in breast adenocarcinoma and the nontumorigenic breast cell lines after treatment with peptide.^18^ Cells were seeded in 96-well plates at a density of 4.0 × 10^4^ cells cm^–2^ and incubated for 24 h at 37 °C and 5% CO_2_. After this period, cells were treated with an aqueous peptide solution at concentrations ranging from 1.0 to 128 μM, and plates were incubated for 24 h at 37 °C and 5% CO_2_. Subsequently, each well received 30 μL of MTT solution (5 μg mL^–1^), and cells were incubated at 37 °C and 5% CO_2_ for 45 min to 1 h. The medium was then removed, and dimethyl sulfoxide (DMSO) was added to each well to solubilize formazan crystals, followed by gentle shaking for 15 min. Finally, cell viability was measured in a microplate reader at 570 nm. All data are shown as the mean ± standard deviation of at least three independent experiments for each group. Graphs were generated using Prism version 8.0. Half-maximal inhibitory concentrations (IC_50_) were calculated using the Probit test with Quest Graph IC_50_ Calculator (AAT Bioquest, Inc., Pleasanton, CA, USA).

### Synergy Assay Using Peptide and Photodynamic Therapy

The synergistic effects of peptides combined with photodynamic therapy (PDT) were evaluated in the human breast cancer cell lines MCF-7 and MDA-MB-231, as well as in the nontumorigenic mammary epithelial cell line MCF-10A.

The peptides IsCT1 and its leader analog AKFK-IsCT1 were tested at concentrations up to 4.0 μM. Synthetic hypericin was initially dissolved in dimethyl sulfoxide (DMSO), and the final DMSO concentration in the cell culture medium was maintained below 1% (v/v) in all experimental conditions. Hypericin was evaluated over a concentration range of 0–10.0 μM. Based on cytotoxicity assays toward nontumorigenic MCF-10A cells, two nontoxic concentrations (0.05 and 0.1 μM) were selected for use in the combination experiments.

Hypericin internalization was allowed to occur for 10 min, followed by photoactivation using orange visible light (λ = 590 nm, 32 mW cm^–2^) for an additional 10 min, as previously described.^71^ Two treatment sequences were employed to investigate the influence of administration order on synergistic effects:

- **Protocol 1:** Hypericin was added first, followed by a 10 min internalization period and subsequent irradiation. Immediately after photoactivation, the peptide solution was added to the cells.
- **Protocol 2:** Cells were initially treated with the peptide solution, followed by the addition of hypericin and completion of its internalization and irradiation steps.

After completion of each treatment protocol, cells were incubated for 4 h at 37 °C in a humidified atmosphere containing 5% CO_2_. Cell viability was then assessed using the MTT assay. Synergistic, additive, or antagonistic interactions between peptides and PDT were quantitatively analyzed using a full dose–response matrix (Table S3) in SynergyFinder 3.0, applying Bliss independence, Loewe additivity, and Highest Single Agent (HSA) reference models.

### *In Viv*o Study

Adult female BALB/c mice were engrafted with 4T1 mouse mammary carcinoma cells (2 × 10^5^ cells). Animals were randomly assigned to experimental groups (n = 5 per group). Mice receive intratumoral injection of saline (control) or peptides (IsCT1 or AKFK-IsCT1 at 1 μM in saline). Treatment began 7 days after tumor cell injection and consisted of 10 applications administered every 2 days. Tumor dimensions were measured with calipers, and volumes were calculated using the formula (L × P^2^)/2, where L is the longest diameter, and P is the perpendicular diameter.

A priori power analysis (G*Power 3.1) was performed to determine the minimum sample size required to detect a large effect size (f = 0.4) with 80% power and α = 0.05 using one-way ANOVA, resulting in a minimum of 5 animals per group. No animals were excluded unless they met predefined humane end point criteria (e.g., ulceration, impaired mobility, or >20% weight loss). The first mouse reaching these criteria triggered euthanasia of all animals in the same experimental set to standardize end point collection. Tumors and spleens were excised and weighed immediately after euthanasia.

Tumor tissue samples were processed and paraffin-embedded for histological analysis. Sections (5 μm) were dewaxed in xylene, rehydrated through graded ethanol, and stained with hematoxylin and eosin (H&E).^72^ Slides were dehydrated, cleared, mounted, and examined under a light microscope (10× and 20× objectives) to assess tissue architecture, cellular morphology, necrosis, inflammatory infiltrates, and tumor response.

After weighing, aliquots of spleen-derived cells and tumor-derived cells were collected under sterile conditions. A density of 10^5^ cells per well was used to seed splenocytes or tumor cells in 24-well plates. Cells were labeled with 5 μL of carboxyfluorescein diacetate succinimidyl ester (CFSE-DA—Thermo Fisher) in PBS 0.1% human albumin, and the wells were filled with DMEM high-glucose supplemented with 10% FBS. Selected wells were treated with Concanavalin A (a positive control for cell proliferation induction) or peptide at their respective IC_50_. Plates were incubated at 37 °C and 5% CO_2_ for 48 h, after which cells were washed, fixed in 1% paraformaldehyde, and stored until analysis.

Flow cytometry was performed on a FACScanto cytometer (BD Bioscience), and data were analyzed using ModFit LT 5.0 to quantify proliferation indices of CFSE-labeled cells. All experiments were performed in three independent biological replicates per animal and cell type.

All procedures involving animals were approved by institutional animal experimentation ethics committees (CEUA) under protocols CEUA/UNIFESP: 8628020620 and CEUAIB: 3558280421 (Figure S4A and B).

### Statistical Analysis

All statistical analyses were performed using GraphPad Prism (version 8.0.2). All data were evaluated for normality before applying statistical tests. Differences between groups were assessed using two-way ANOVA followed by Tukey’s post hoc test, and results were considered statistically significant at *p* < 0.05. Whenever appropriate and consistent with ethical guidelines, multiple tissues, organs, or whole blood samples from the same animal were used for different measurements to minimize animal use (e.g., distinct parameters in whole blood were obtained from the same specimens).

## Supplementary Material



## Notes

Additional experimental data and analyses that complement the main manuscript, comprises chromatograms and mass spectra of peptides, complete dose–response matrices, supplementary synergy analyses using the Loewe and HSA models, additional cell viability assays, expanded statistical analyses, and supplementary figures illustrating protocol-dependent effects of peptide–PDT combinations; hematological and immunological parameter evaluations to support the interpretation of the antitumor and immunomodulatory effects of IsCT1 and AKFK-IsCT1 (PDF)

## Abbreviations

CD: Circular dichroism
CFSE-DA: Carboxyfluorescein diacetate succinimidyl ester
DMSO: Dimethyl sulfoxide
ERBB2: Tyrosine kinase 2 receptor (HER2)
Fmoc: Fluoromethyloxycarbonyl
FSB: Fetal serum bovine
H&E: Hematoxylin and eosin staining
HDP: Host defense peptide
Hyp: Hypericin
HR^+^: Hormone receptor-positive
IC_50_: Half-maximal inhibitory concentration
LC/ESI-MS: Liquid chromatography electrospray ionization mass spectrometry
MTT: 3-(4,5-Dimethylthiazol-2-yl)-2,5-diphenyltetrazolium bromide
PBS: Phosphate-buffered saline
PDT: Photodynamic therapy
PDT-Hyp: Photodynamic therapy using Hyp as PS
PS: Photosensitizer
ROS: Reactive oxygen species
RP-HPLC: Reverse-phase high-performance liquid chromatography
SDS: Sodium dodecyl sulfate
TFE: Trifluoroethanol
TI: Therapeutic index
TNBC: Triple-negative breast cancer
WBC: White blood cell
