# Supplementary material for: Engineering the Host Defense Peptide from Scorpion Venom for Safer and More Potent Antibreast Cancer Activity
Source: ACS Pharmacol Transl Sci. 2026 May 5;9(6):1388–99. doi: 10.1021/acsptsci.5c00598 (PMC13270443; doi:10.1021/acsptsci.5c00598)
Supplement: Supplementary file 1 [file pt5c00598_si_001.pdf]

SUPPORTING INFORMATION

Engineering the Host Defense Peptide from Scorpion Venom for Safer and More Potent Anti-Breast Cancer Activity

Cyntia Silva Oliveira [1,2,5*], Laertty Garcia Sousa Cabral [3,4], Rosely Cabette Barbosa Alves [4], Gislaine Patricia Andrade [2,5,6], Anderson Orzari Ribeiro [5], Kaio Moraes Farias [4], Durvanei Augusto Maria [3,4], Giselle Cerchiaro [5], Vani Xavier Oliveira Jr. [2,5*].

[1] Laboratory of Viral Biotechnology, Butantan Institute, São Paulo, 05508-210, Brazil.
[2] Paulista School of Medicine, Postgraduate Program in Molecular Biology, Federal University of São Paulo, São Paulo 04044-020, Brazil.
[3] Faculty of Medicine, University of São Paulo, São Paulo 05508-220, Brazil.
[4] Laboratory of Development and Innovation, Butantan Institute, São Paulo 05585-000, Brazil.
[5] Center for Natural and Human Sciences, Federal University of ABC, Santo Andre 09280-560, Brazil.
[6] Biochemistry Department, University Center FMABC, Santo André 09060-650, Brazil.
*Correspondence: Vani Xavier Oliveira Jr. (vani.junior@ufabc.edu.br); Cyntia Silva Oliveira (c.oliveira.proppg@propp.butantan.gov.br)

Supporting Information Index

Figure S 1. Chromatograms and mass spectrometry of IsCT1 and analogs. 2
Figure S 2. Cell viability following combined treatment with PDT-Hyp and peptides. ...16
Figure S 3. Heatmap representation of Bliss synergy scores. 17
Table S 1. The pharmacological effect of different protocols combining PDT-Hyp with peptides.. 18
Table S 2. Hematological parameters of animals before tumor cell inoculation and after treatment.. 18
Table S 3. Dose–response matrix for the combined treatment of photodynamic therapy with hypericin (PDT-Hyp) and peptide...19

Figure S 1. Chromatograms and mass spectrometry of IsCT1 and analogs. A) Chromatogram of IsCT1. B) Mass spectrometry of IsCT1. C) Chromatogram of P-IsCT1. D) Mass spectrometry of P-IsCT1. E) Chromatogram of KP-IsCT1. F) Mass spectrometry of KP-IsCT1. G) Chromatogram of AKFK-IsCT1. H) Mass spectrometry of AKFK-IsCT1. I) Chromatogram of AFPK-IsCT1. J) Mass spectrometry of AFPK-IsCT1. K) Chromatogram of KKK-IsCT1. L) Mass spectrometry of KKK-IsCT1. M) Chromatogram of KKPK-IsCT1. N) Mass spectrometry of KKPK-IsCT1.

A

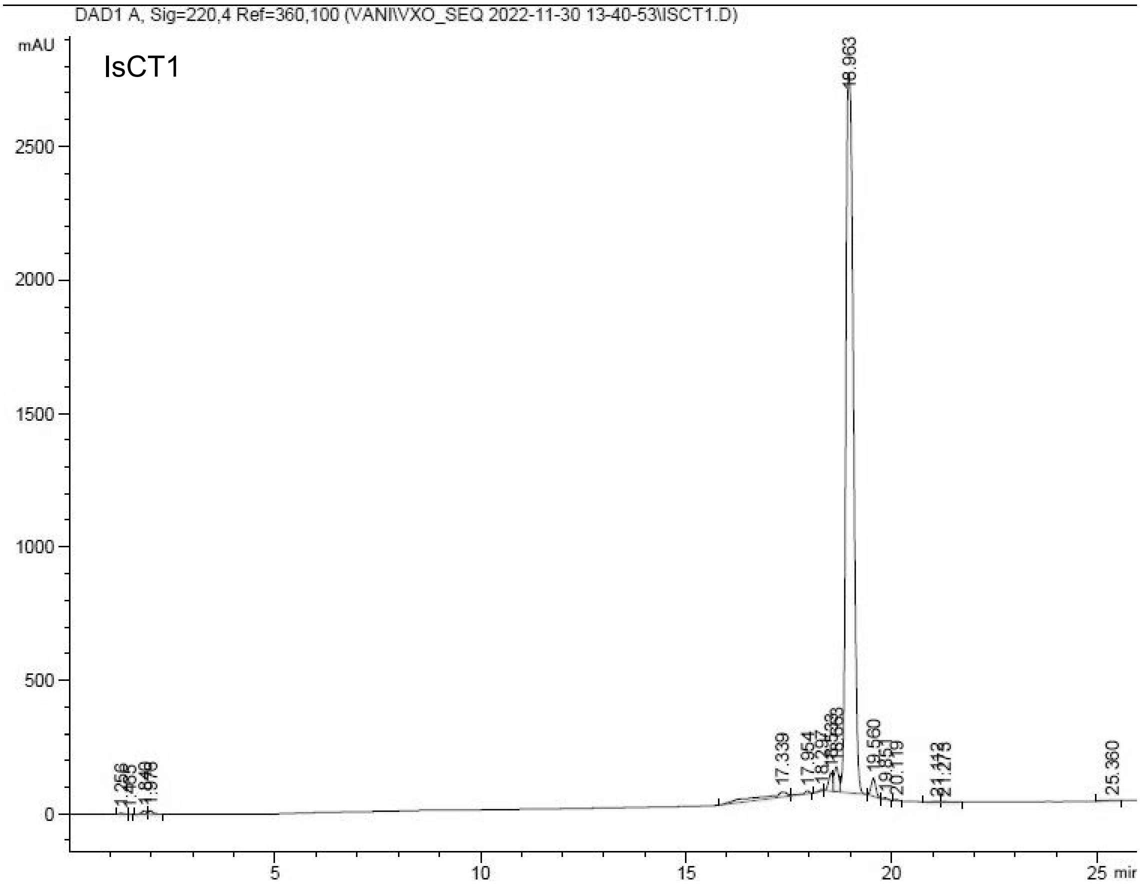

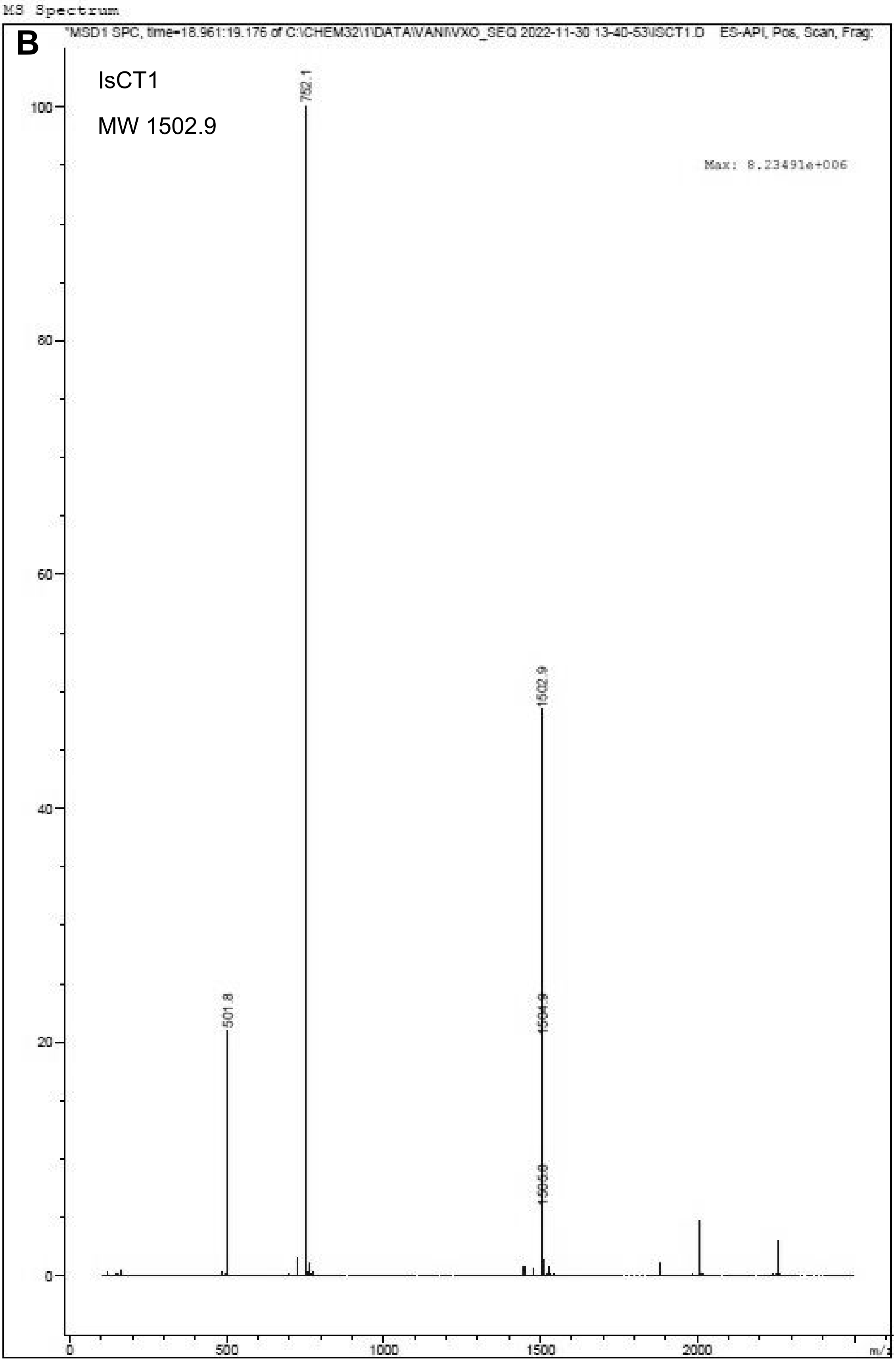
MS Spectrum
B
*MSD1 SPC, time=18.961:19.176 of C:\CHEM32\1\DATA\VANI\VXO_SEQ 2022-11-30 13-40-53\ISCT1.D ES-API, Pos, Scan, Frag:
IsCT1
MW 1502.9
Max: 8.23491e+006
752.1
1502.9
501.8
100
80
60
40
20
0
0
500
1000
1500
2000
m/z

C

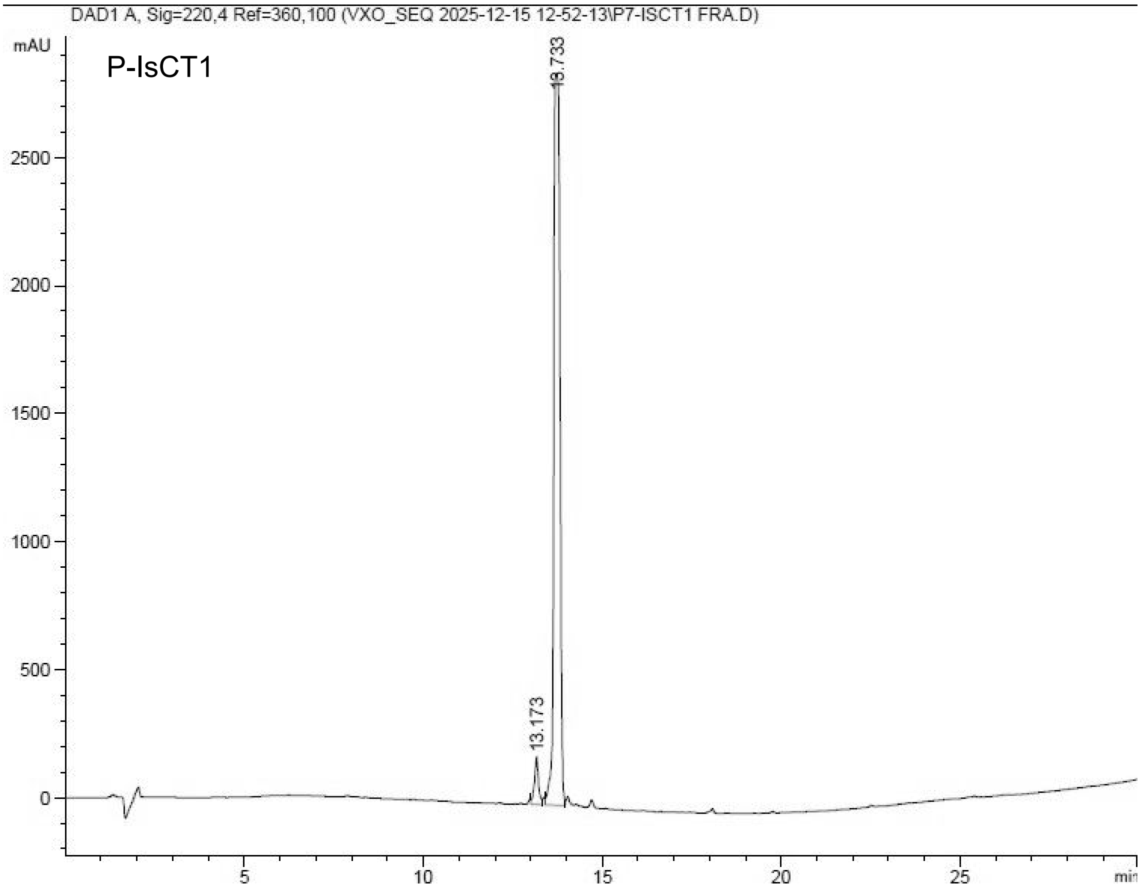
DAD1 A, Sig=220,4 Ref=360,100 (VXO_SEQ 2025-12-15 12-52-13\P7-ISCT1 FRA.D)
mAU
P-IsCT1
13.733
13.173
2500
2000
1500
1000
500
0
5
10
15
20
25
min

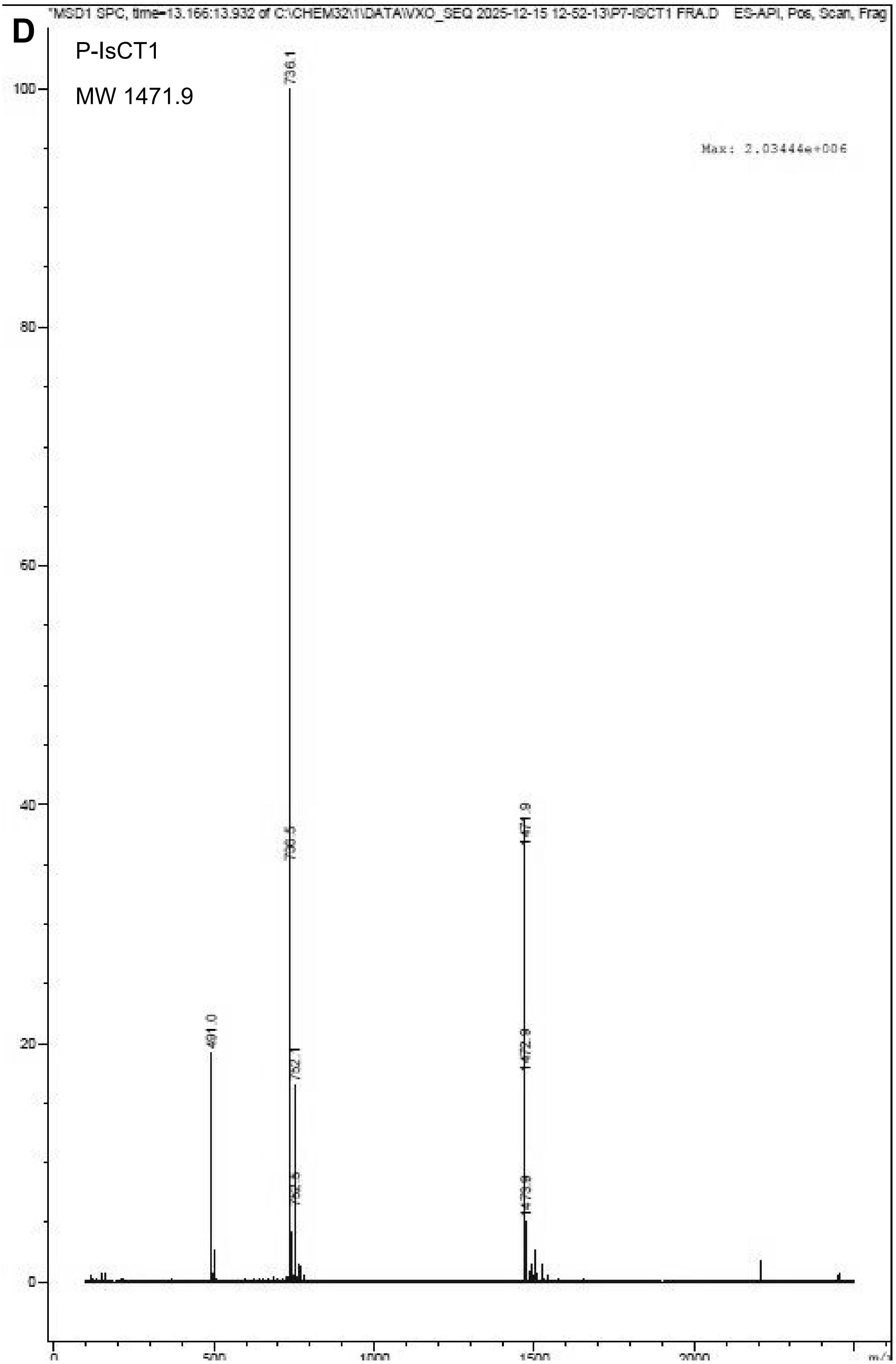
*MSD1 SPC, time=13.166:13.932 of C:\CHEM32\1\DATA\VXO_SEQ 2025-12-15 12-52-13\P7-ISCT1 FRA.D ES-API, Pos, Scan, Frag
D
P-IsCT1
MW 1471.9
Max: 2.03444e+006
736.1
736.5
752.1
752.5
491.0
1471.9
1472.9
1473.9
100
80
60
40
20
0
0
500
1000
1500
2000

E

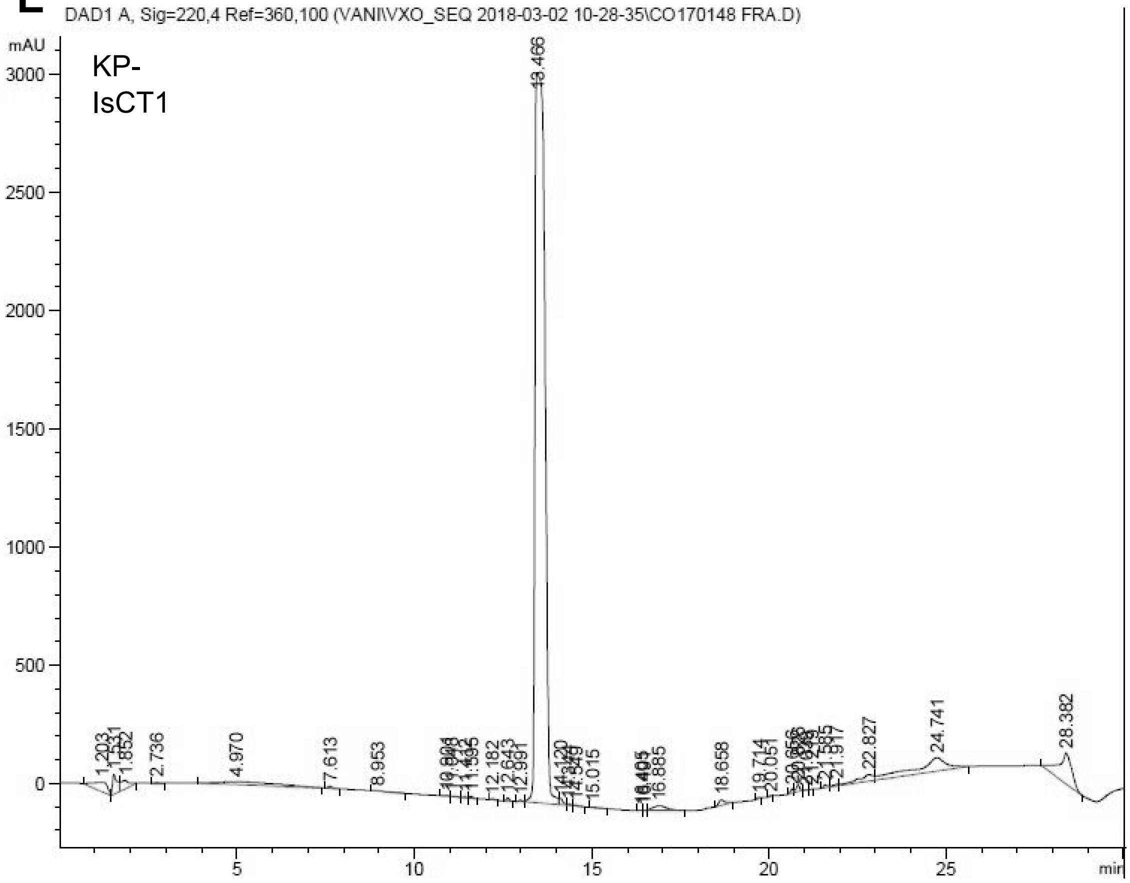
DAD1 A, Sig=220,4 Ref=360,100 (VANI\VXO_SEQ 2018-03-02 10-28-35\CO170148 FRA.D)
mAU
KP-
IsCT1
13.466
24.741
28.382
22.827
min

F

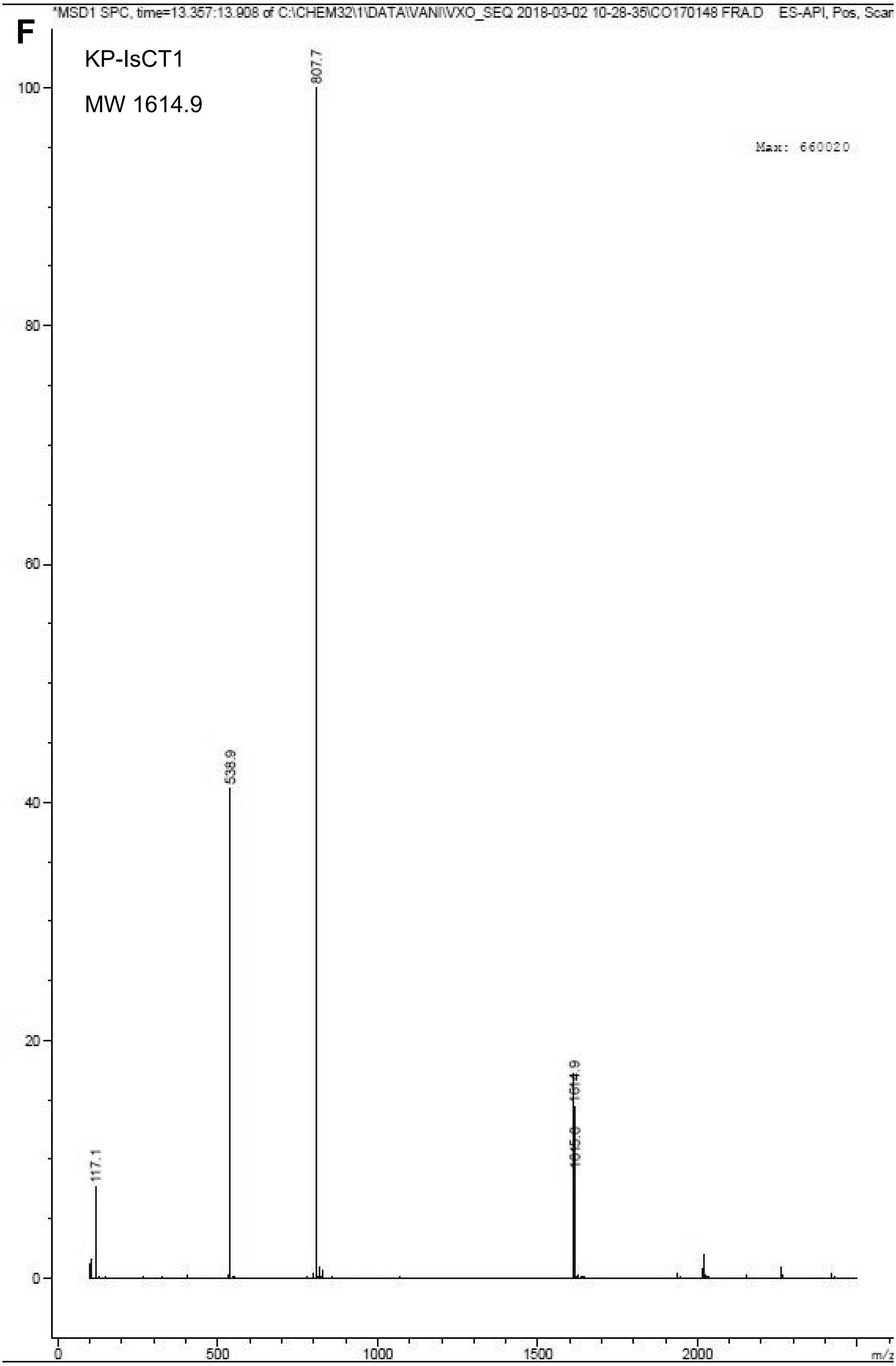

*MSD1 SPC, time=13.357:13.908 of C:\CHEM32\1\DATA\VANI\VXO_SEQ 2018-03-02 10-28-35\CO170148 FRA.D ES-API, Pos, Scan
KP-IsCT1
MW 1614.9
Max: 660020
807.7
538.9
1614.9
1615.9
117.1
100
80
60
40
20
0
0
500
1000
1500
2000
m/z

G

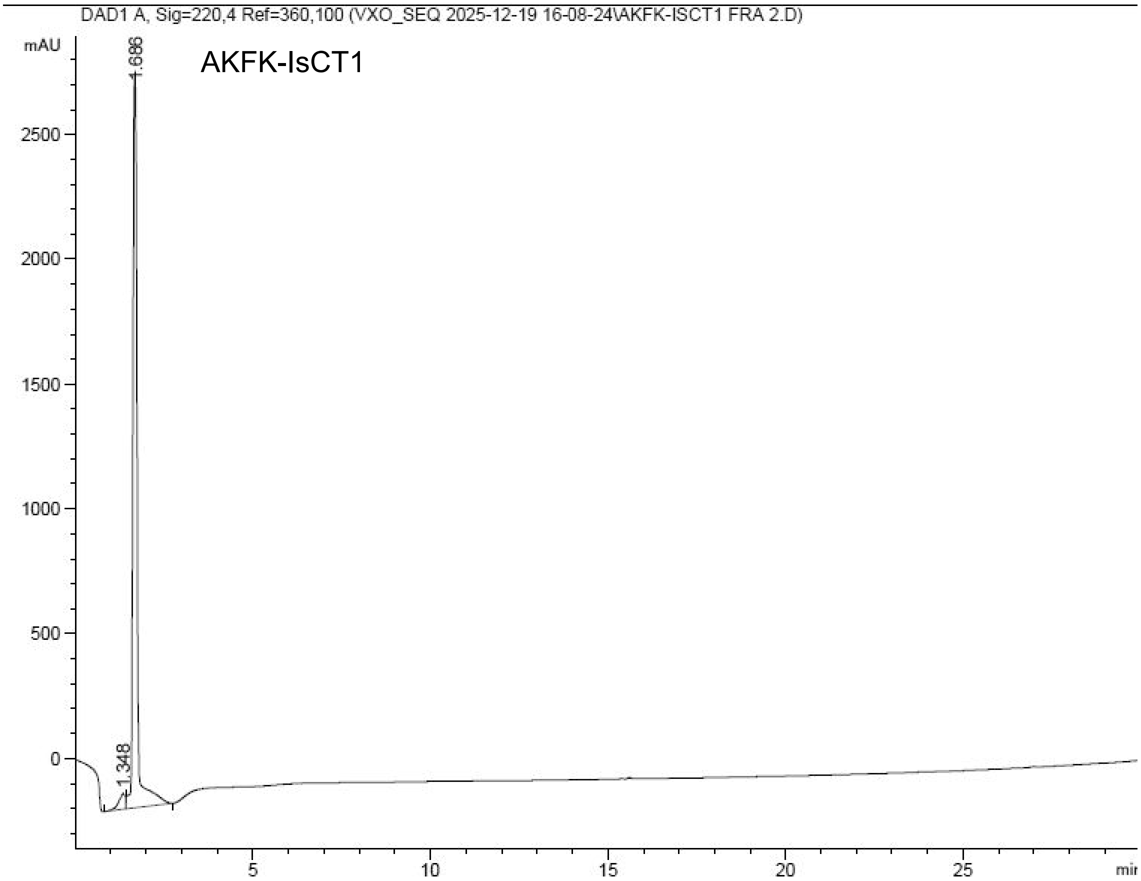
DAD1 A, Sig=220,4 Ref=360,100 (VXO_SEQ 2025-12-19 16-08-24\AKFK-ISCT1 FRA 2.D)
mAU
AKFK-IsCT1
1.686
1.348
2500
2000
1500
1000
500
0
5
10
15
20
25
mir

H

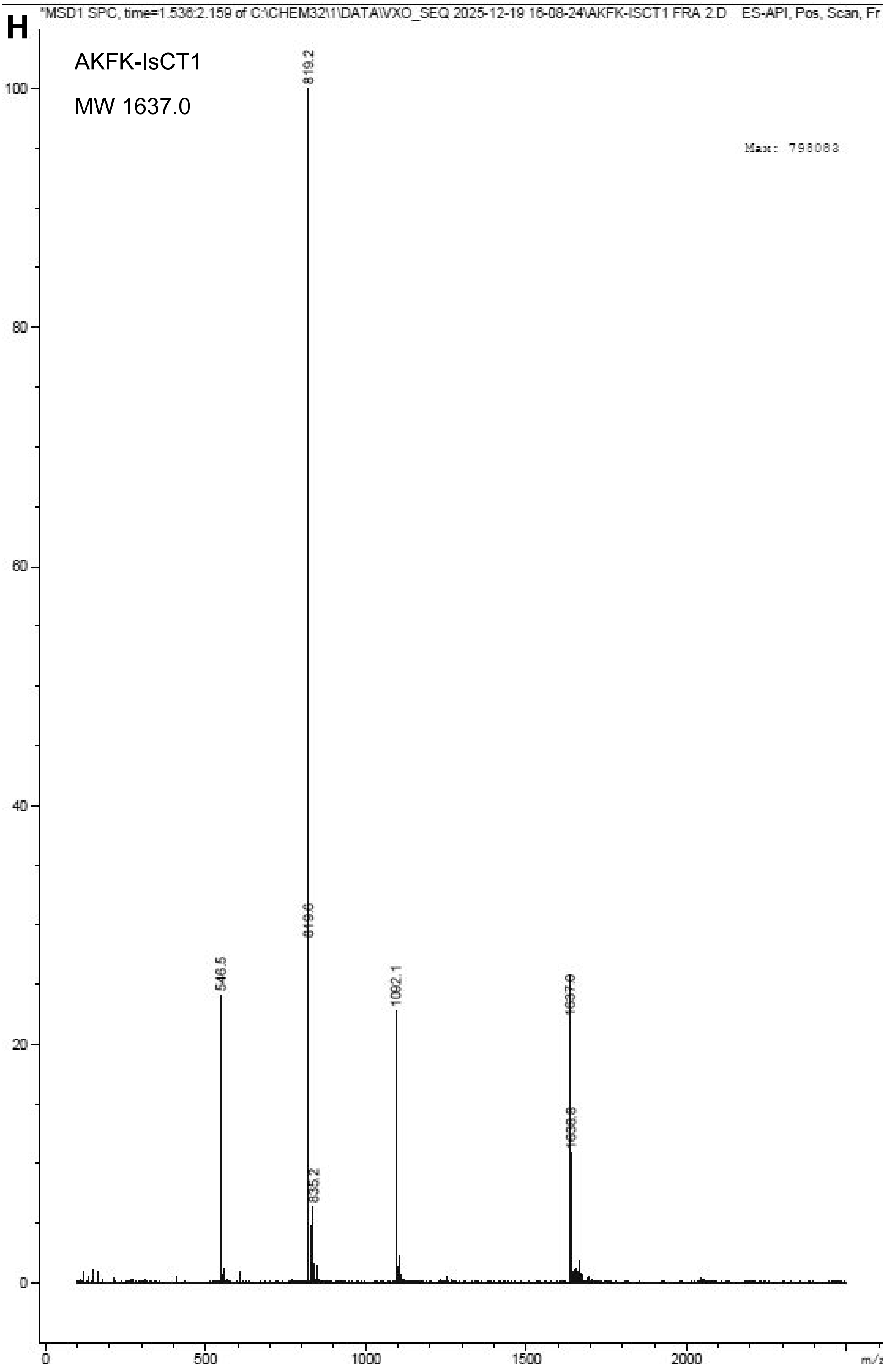
*MSD1 SPC, time=1.536:2.159 of C:\CHEM32\1\DATA\VXO_SEQ 2025-12-19 16-08-24\AKFK-ISCT1 FRA 2.D ES-API, Pos, Scan, Fr
AKFK-IsCT1
MW 1637.0
Max: 798083
819.2
546.5
1092.1
835.2
100
80
60
40
20
0
500
1000
1500
2000
m/z

I

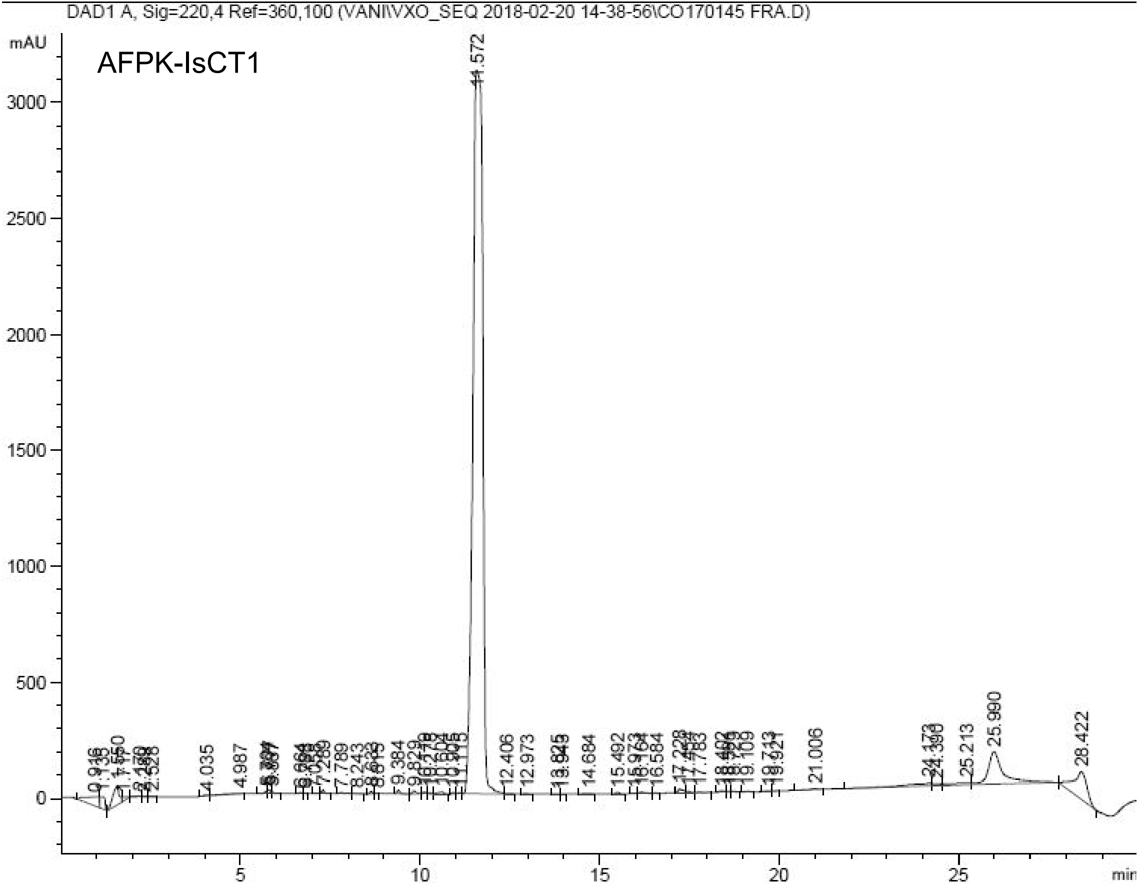
DAD1 A, Sig=220,4 Ref=360,100 (VANI\VXO_SEQ 2018-02-20 14-38-56\CO170145 FRA.D)
mAU
AFPK-IsCT1
11.572
25.990
28.422
min

J

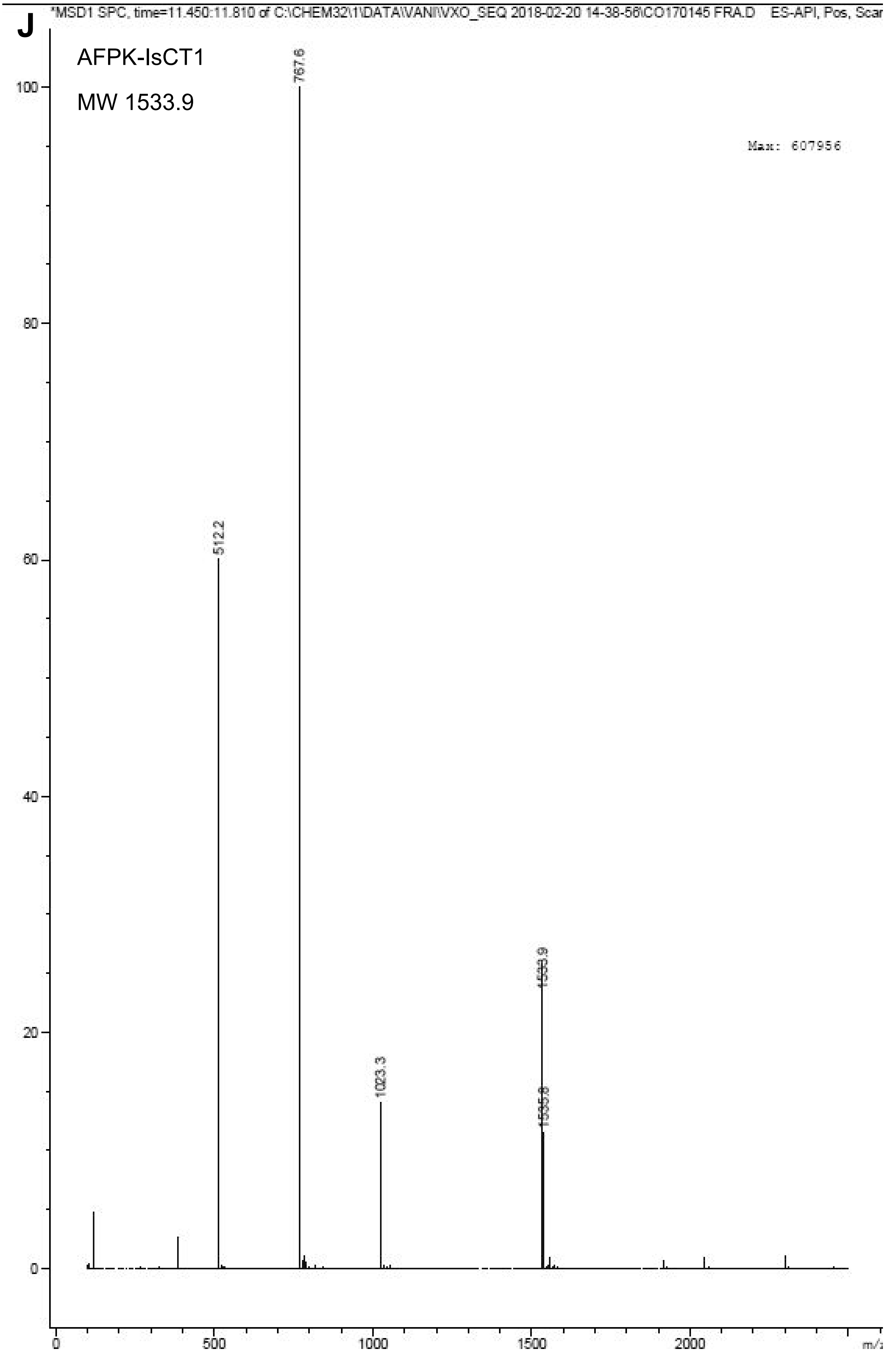
*MSD1 SPC, time=11.450:11.810 of C:\CHEM32\1\DATA\VANI\VXO_SEQ 2018-02-20 14-38-56\CO170145 FRA.D ES-API, Pos, Scan
AFPK-IsCT1
MW 1533.9
Max: 607956
767.6
512.2
1023.3
1533.9
1535.8
0
20
40
60
80
100
0
500
1000
1500
2000
m/z

K

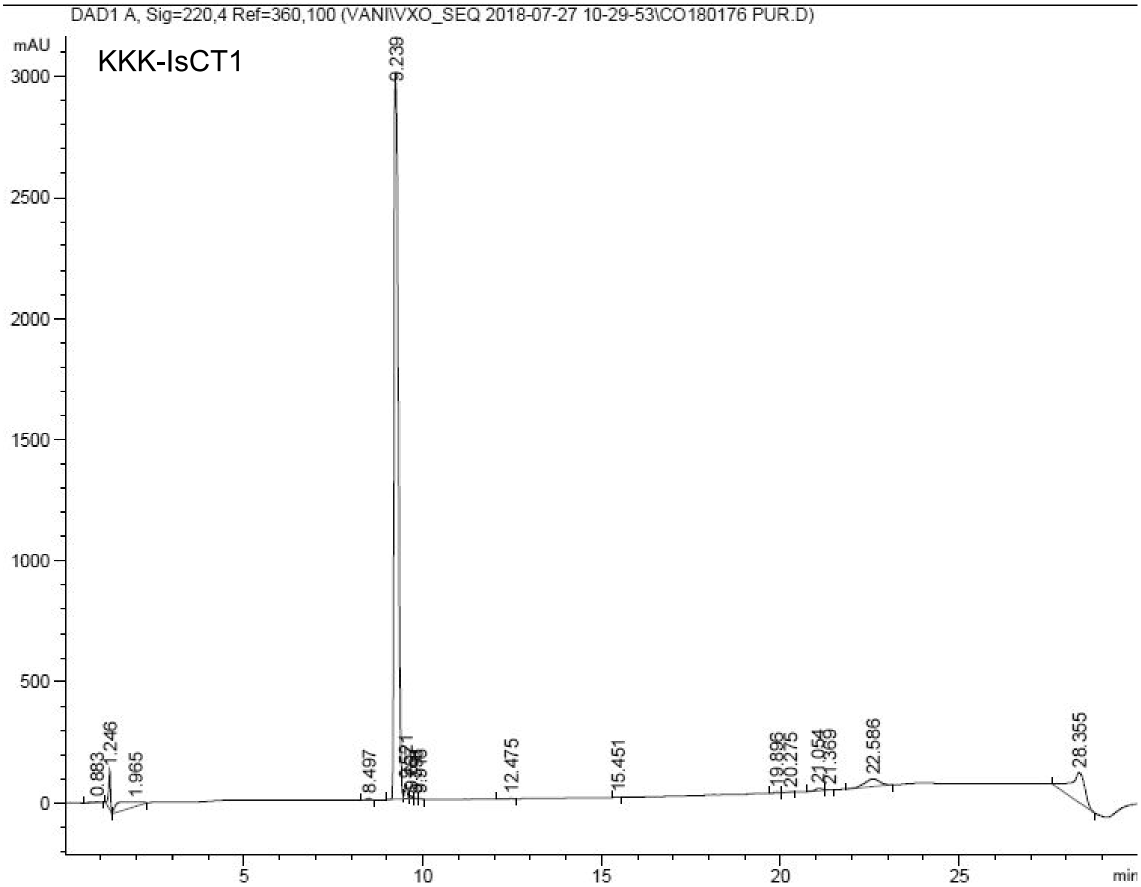
DAD1 A, Sig=220,4 Ref=360,100 (VANI\VXO_SEQ 2018-07-27 10-29-53\CO180176 PUR.D)
mAU
KKK-IsCT1
9.239
0.883
1.246
1.965
8.497
12.475
15.451
22.586
28.355
min

L

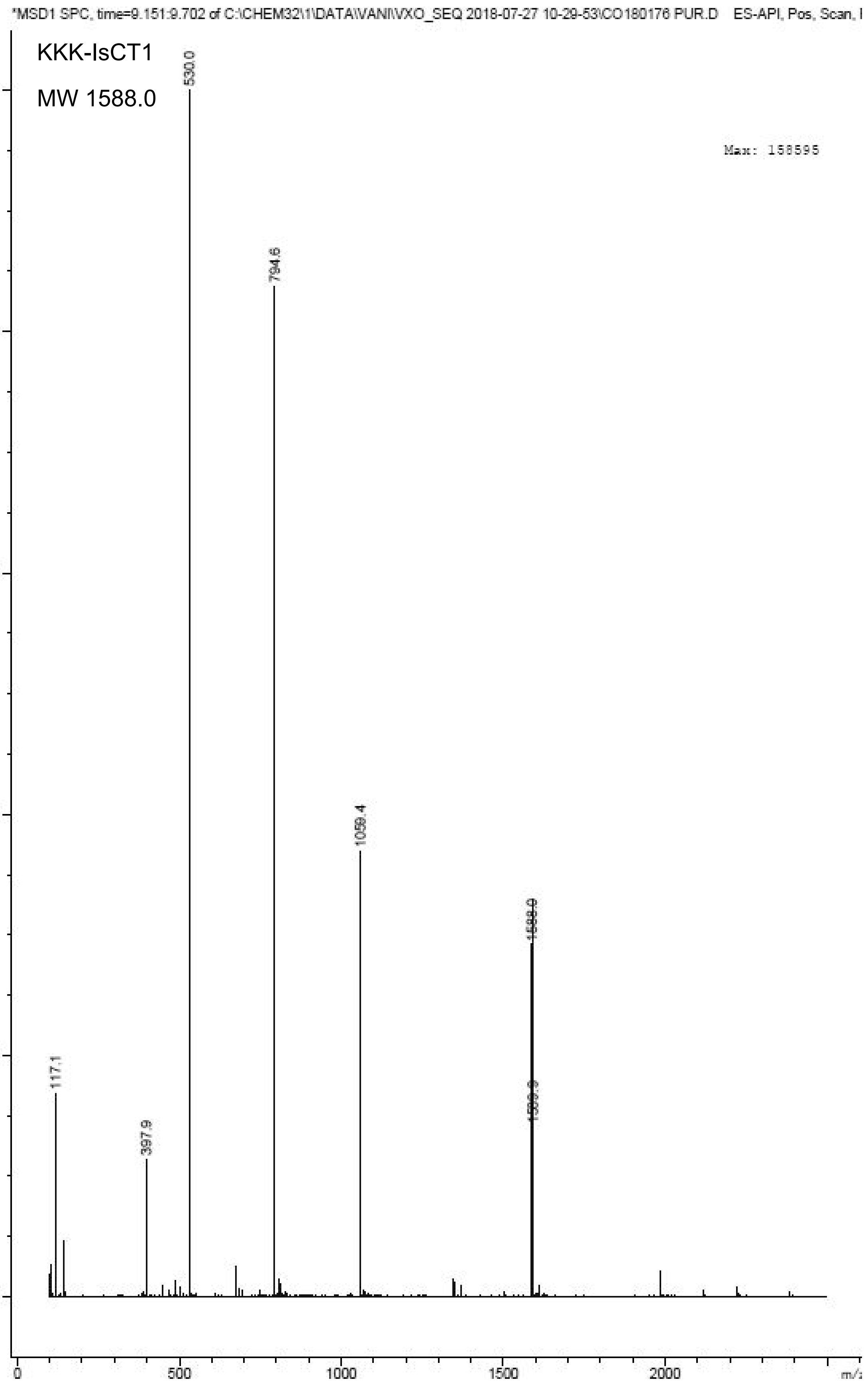
*MSD1 SPC, time=9.151:9.702 of C:\CHEM32\1\DATA\VANI\VXO_SEQ 2018-07-27 10-29-53\CO180176 PUR.D ES-API, Pos, Scan, I
KKK-IsCT1
MW 1588.0
Max: 158595
530.0
794.6
1059.4
1588.0
117.1
397.9
100
80
60
40
20
0
0
500
1000
1500
2000
m/z

M

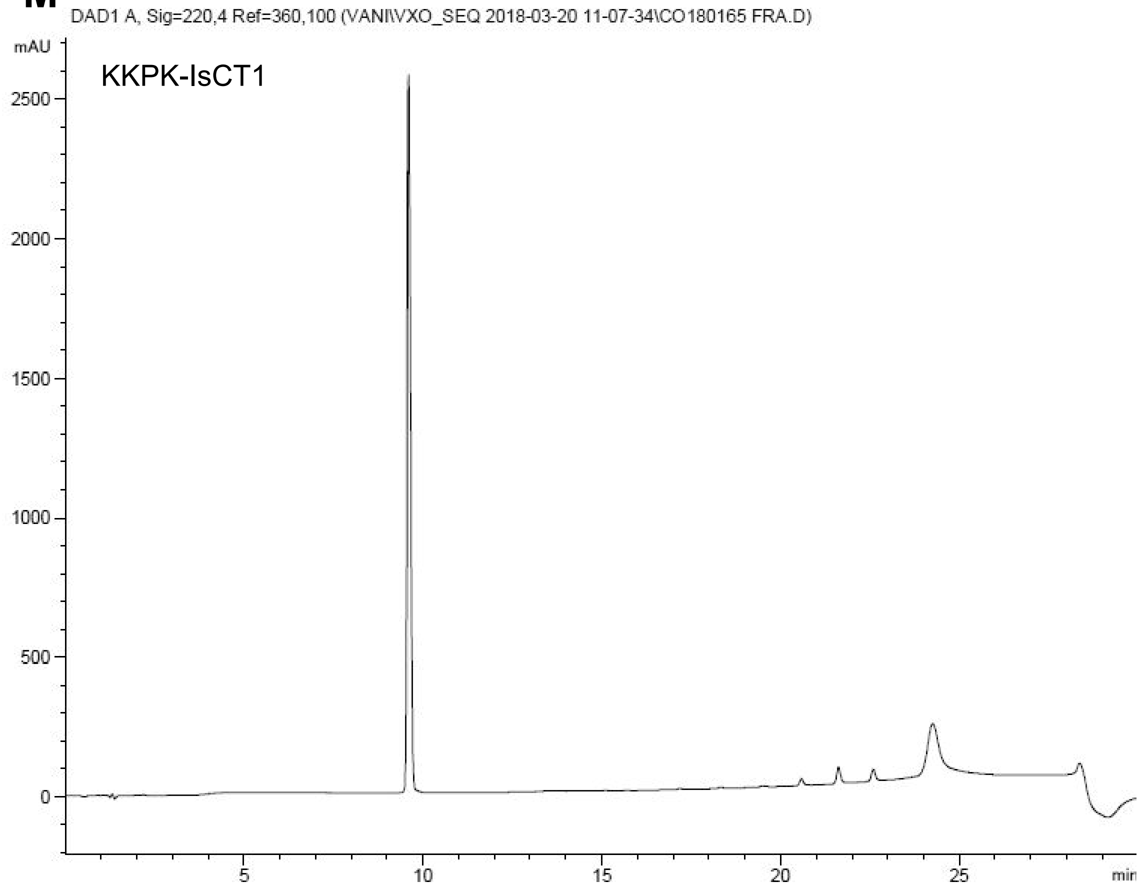
DAD1 A, Sig=220,4 Ref=360,100 (VANI\VXO_SEQ 2018-03-20 11-07-34\CO180165 FRA.D)
mAU
KKPK-IsCT1
2500
2000
1500
1000
500
0
5
10
15
20
25
min

N

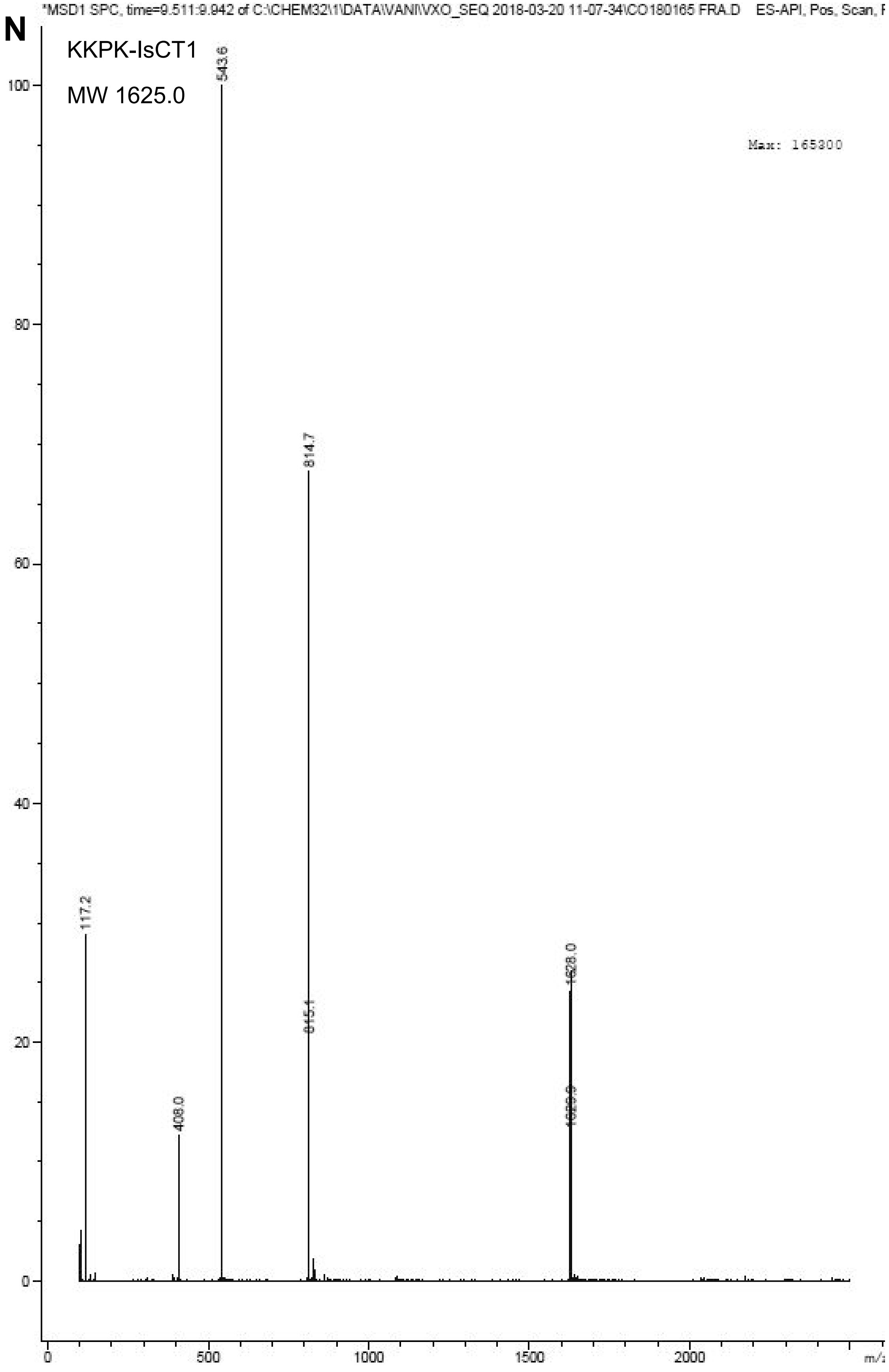
*MSD1 SPC, time=9.511:9.942 of C:\CHEM32\1\DATA\VANI\VXO_SEQ 2018-03-20 11-07-34\CO180165 FRA.D ES-API, Pos, Scan, F
KKPK-IsCT1
MW 1625.0
Max: 165300
543.6
814.7
117.2
1628.0
815.1
408.0
m/z

Figure S 2. Cell viability following combined treatment with PDT-Hyp (0.05 or 0.1 µM) and peptides. Treatments were applied using Protocol 1 in (A) MCF-10A, (B) MCF-7, and (C) MDA-MB-231 cells, and Protocol 2 in (D) MCF-10A, (E) MCF-7, and (F) MDA-MB-231 cells. Blue and green bars indicate IsCT1 combined with PDT-Hyp at 0.05 and 0.1 µM, respectively, whereas orange and pink bars indicate AKFK-IsCT1 combined with PDT-Hyp at 0.05 and 0.1 µM, respectively. Bright colors denote Protocol 1, and soft colors denote Protocol 2. Peptides were tested at concentrations ranging from 0.0 to 4.0 µM, followed by incubation at 37 °C and 5% CO_2 for 4 h. Data represent the mean of three independent experiments performed in triplicate.

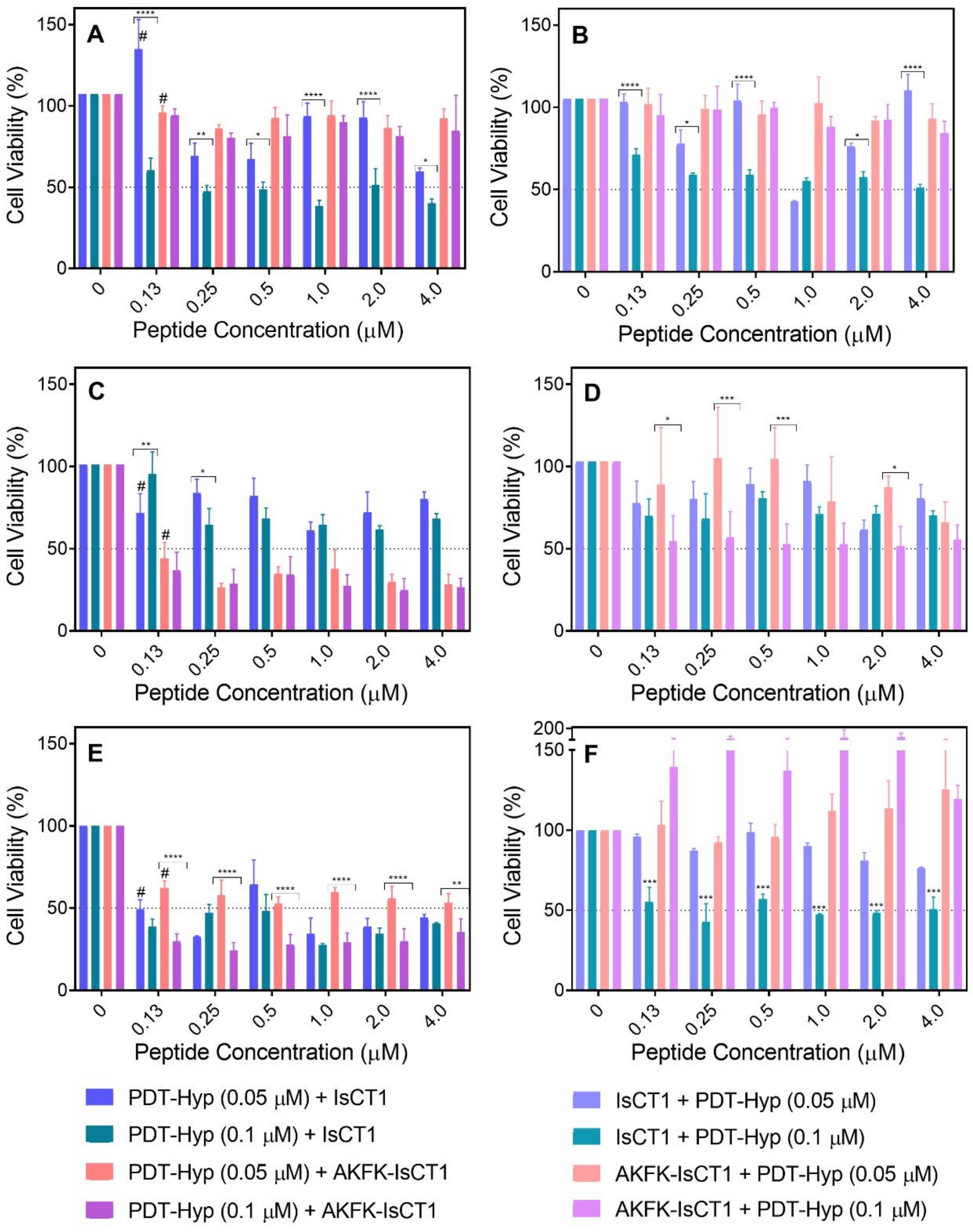

Figure S 3. Heatmap representation of Bliss synergy scores. Data were obtained from complete dose–response matrices for the combined treatment of photodynamic therapy with hypericin (PDT-Hyp) and the peptides IsCT1 or AKFK-IsCT1. Protocol 1 was evaluated with IsCT1 (A, E, I) or AKFK-IsCT1 (C, G, K), whereas Protocol 2 was evaluated with IsCT1 (B, F, J) or AKFK-IsCT1 (D, H, L). Panels in the first row (A–D) correspond to MCF-10A cells, the second row (E–H) to MCF-7 cells, and the third row (I–L) to MDA-MB-231 cells. Analyses were performed using SynergyFinder 3.0. Color gradients indicate the magnitude and nature of the interaction, with green representing antagonistic effects (≤ 0), intermediate colors indicating additive effects (> 0 and ≤ 10), and red representing synergistic interactions (> 10). Data correspond to the same experimental conditions shown in the main figures and are presented to provide a complementary visualization of the synergy landscape.

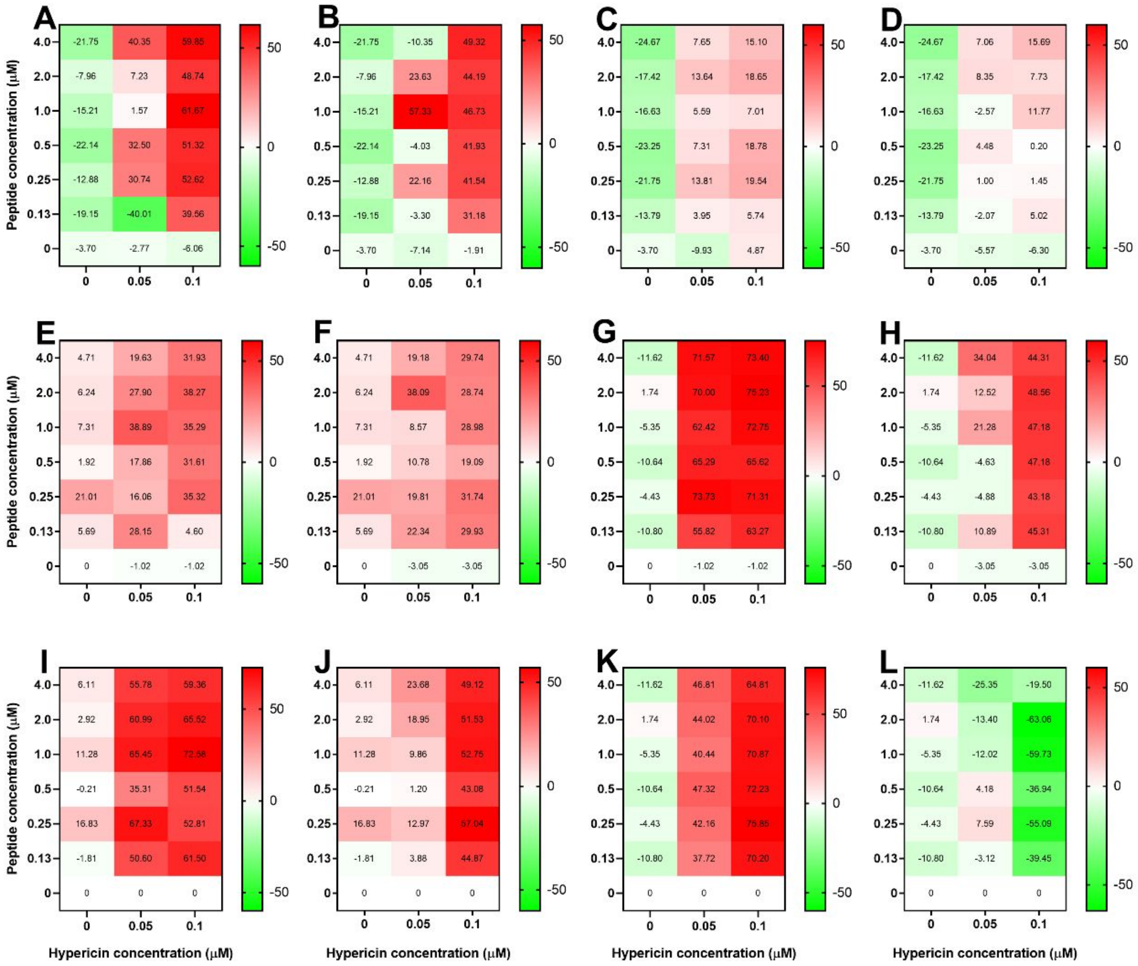

Table S 1. The pharmacological effect of different protocols combining PDT-Hyp with peptides. Treatments were applied against non-tumorigenic (MCF-10A) and breast cancer cell lines (MCF-7, MDA-MB-231, and 4T1). Were showed synergy scores by Bliss, Loewe additivity and Highest Single Agent (HSA) models.

Cell line	Combination[a]	Protocol	Bliss Synergy Score	HSA Synergy Score	Lowe Synergy Score	Effect
MCF-10A	PDT-Hyp+IsCT1	1	28.17	30.54	31.94	Synergy
	IsCT1+ PDT-Hyp	2	25.07	27.46	25.97	Synergy
	PDT-Hyp +AKFK-IsCT1	1	6.8	11.04	5.55	Additive
	AKFK-IsCT1+ PDT-Hyp	2	4.16	8.54	8.83	Additive
MCF-7	PDT-Hyp+IsCT1	1	19.1	19.11	18.91	Synergy
	IsCT1+ PDT-Hyp	2	15.53	15.61	15.59	Synergy
	PDT-Hyp +AKFK-IsCT1	1	64.21	64.21	64.64	Synergy
	AKFK-IsCT1+ PDT-Hyp	2	29.19	28.88	31.28	Synergy
MDA-MB-231	PDT-Hyp+IsCT1	1	52.37	52.04	52.32	Synergy
	IsCT1+ PDT-Hyp	2	24.88	24.55	24.84	Synergy
	PDT-Hyp +AKFK-IsCT1	1	52.56	52.72	52.99	Synergy
	AKFK-IsCT1+ PDT-Hyp	2	-24.95	-24.79	-13.88	Antagonist

Table S 2. Hematological parameters of animals before tumor cell inoculation and after treatment. Total and differential white blood cell counts were determined in peripheral blood collected before tumor cell inoculation (pre-inoculation) and after ectopic tumor induction in animals treated with vehicle (control), IsCT1, or AKFK-IsCT1. Data are presented as mean ± standard deviation and expressed as $\times 10^3$ cells μL^{-1}. NA indicates not available. Statistical significance was determined relative to the control group (*$p < 0.05$; **$p < 0.01$).

White blood cells	Pre-inoculation[1]	Control	IsCT1	AKFK-IsCT1
Leukocytes ($10^3/\mu L$)	6.5 ± 1.2*	47,0 ± 12,5	40,2 ± 26,0	71,1 ± 42,7*
Neutrophil ($10^3/\mu L$)	0.9 ± 0.2	2,8 ± 0,5	2,8 ± 1,9	7,0 ± 5,3
Lymphocytes ($10^3/\mu L$)	5.4 ± 1.0**	42,0 ± 11,0	35,1 ± 21,6	55,9 ± 30,1**
Monocytes ($10^3/\mu L$)	NA	1,6 ± 0,7	1,6 ± 1,8	6,1 ± 5,4
Eosinophil ($10^3/\mu L$)	NA	0,06 ± 0,03	0,04 ± 0,03	0,1 ± 0,1
Basophil ($10^3/\mu L$)	NA	0,5 ± 0,2	0,7 ± 0,7	2,0 ± 1,9
Atypical lymphocyte ($10^3/\mu L$)	NA	0,9 ± 0,4	1,0 ± 1,1	2,4 ± 2,0
Immature cells (%)	NA	0,06 ± 0,04	0,1 ± 0,2	0,8 ± 0,8

1 – Pre-inoculation: data media of all animals before 4T1 cells inoculation; *p<0.04; **p<0.001. NA: not available

Table S 3. Dose–response matrix for the combined treatment of photodynamic therapy with hypericin (PDT-Hyp) and peptide. Cells were treated with increasing concentrations of hypericin (rows) and peptide (columns) under the indicated treatment protocol. Each matrix cell represents the mean percentage of cell viability relative to untreated controls, obtained from three independent experiments performed in triplicate. These matrices constitute the complete dose–response datasets used for synergy analysis and were used as input for Bliss, Loewe, and Highest Single Agent (HSA) models in SynergyFinder 3.0.

**MCF-10A**																
Protocol 1									Protocol 2							
	IsCT1 (μM)									IsCT1 (μM)						
Hyp (μM)	0	0.13	0.25	0.5	1	2	4		Hyp (μM)	0	0.13	0.25	0.5	1	2	4
0	103.70	119.15	112.88	122.14	115.21	107.96	121.75		0.00	103.70	119.15	112.88	122.14	115.21	107.96	121.75
0.05	102.77	140.01	69.26	67.50	98.43	92.77	59.65		0.05	107.14	103.30	77.84	104.03	42.67	76.37	110.35
0.1	106.06	60.44	47.38	48.68	38.33	51.26	40.15		0.10	101.91	68.82	58.46	58.07	53.27	55.81	50.68
	AKFK-IsCT1 (μM)									AKFK-IsCT1 (μM)						
Hyp (μM)	0	0.13	0.25	0.5	1	2	4		Hyp (μM)	0	0.13	0.25	0.5	1	2	4
0	103.70	113.79	121.75	123.25	116.63	117.42	124.67		0.00	103.70	113.79	121.75	123.25	116.63	117.42	124.67
0.05	109.93	96.05	86.19	92.69	94.41	86.36	92.35		0.05	105.57	102.07	99.00	95.52	102.57	91.65	92.94
0.1	95.13	94.26	80.46	81.22	92.99	81.35	84.90		0.10	106.30	94.98	98.55	99.80	88.23	92.27	84.31
**MCF-7**																
Protocol 1									Protocol 2							
	IsCT1 (μM)									IsCT1 (μM)						
Hyp (μM)	0	0.13	0.25	0.5	1	2	4		Hyp (μM)	0	0.13	0.25	0.5	1	2	4
0	100.00	94.31	78.99	98.08	92.69	93.76	95.29		0.00	100.00	94.31	78.99	98.08	92.69	93.76	95.29
0.05	101.02	71.85	83.94	82.14	61.11	72.10	80.37		0.05	103.05	77.66	80.19	89.22	91.43	61.91	80.82
0.1	101.02	95.40	64.68	68.39	64.71	61.73	68.07		0.10	103.05	70.07	68.26	80.91	71.02	71.26	70.26
	AKFK-IsCT1 (μM)									AKFK-IsCT1 (μM)						
Hyp (μM)	0	0.13	0.25	0.5	1	2	4		Hyp (μM)	0	0.13	0.25	0.5	1	2	4
0	100.00	110.80	104.43	110.64	105.35	98.26	111.62		0.00	100.00	110.80	104.43	110.64	105.35	98.26	111.62
0.05	101.02	44.18	26.27	34.71	37.58	30.00	28.43		0.05	103.05	89.11	104.88	104.63	78.72	87.48	65.96

0.1	101.02	36.73	28.69	34.38	27.25	24.77	26.60		0.10	103.05	54.69	56.82	52.82	52.82	51.44	55.69
MDA-MB-231																
Protocol 1									Protocol 2							
	IsCT1 (μM)									IsCT1 (μM)						
Hyp (μM)	0	0.13	0.25	0.5	1	2	4		Hyp (μM)	0	0.13	0.25	0.5	1	2	4
0	100.00	101.81	83.18	100.21	88.73	97.08	93.89		0.00	100.00	101.81	83.18	100.21	88.73	97.08	93.89
0.05	100.00	49.40	32.67	64.69	34.55	39.01	44.22		0.05	100.00	96.12	87.03	98.80	90.14	81.05	76.32
0.1	100.00	38.50	47.19	48.46	27.42	34.48	40.64		0.10	100.00	55.13	42.96	56.92	47.25	48.47	50.88
	AKFK-IsCT1 (μM)									AKFK-IsCT1 (μM)						
Hyp (μM)	0	0.13	0.25	0.5	1	2	4		Hyp (μM)	0	0.13	0.25	0.5	1	2	4
0	100.00	110.80	104.43	110.64	105.35	98.26	111.62		0.00	100.00	110.80	104.43	110.64	105.35	98.26	111.62
0.05	100.00	62.28	57.84	52.68	59.56	55.98	53.19		0.05	100.00	139.45	155.09	136.94	159.73	163.06	119.50
0.1	100.00	29.80	24.15	27.77	29.13	29.90	35.19		0.10	100.00	139.45	155.09	136.94	159.73	163.06	119.50
